# Seasonal migration patterns of Siberian Rubythroat (*Calliope calliope*) facing the Qinghai–Tibet Plateau

**DOI:** 10.1186/s40462-024-00495-5

**Published:** 2024-08-01

**Authors:** Tianhao Zhao, Wieland Heim, Raphaël Nussbaumer, Mariëlle van Toor, Guoming Zhang, Arne Andersson, Johan Bäckman, Zongzhuang Liu, Gang Song, Magnus Hellström, Jacob Roved, Yang Liu, Staffan Bensch, Bregje Wertheim, Fumin Lei, Barbara Helm

**Affiliations:** 1 Groningen Institute for Evolutionary Life Sciences (GELIFES), University of Groningen, 9747 AG Groningen, The Netherlands; 2 Institute of Biology and Environmental Sciences (IBU), Carl von Ossietzky University of Oldenburg, Ammerländer Heerstraße 114-118, 26129 Oldenburg, Germany; 3 Department of Bird Migration, Swiss Ornithological Institute, 6204 Sempach, Switzerland; 4 Department of Evolutionary Biology and Environmental Studies, University of Zurich, Zurich, Switzerland; 5 Cornell Lab of Ornithology, Ithaca, NY USA; 6 Center for Ecology and Evolution in Microbial Model Systems (EEMiS), Linnaeus University, 391 82 Kalmar, Sweden; 7 Qinghai University, Xining, China; 8 Department of Biology, Lund University, 223 62 Lund, Sweden; 9 Department of Ecology and Genetics, Uppsala University, 752 36 Uppsala, Sweden; 10 Key Laboratory of Animal Biodiversity Conservation and Integrated Pest Management, Institute of Zoology, Chinese Academy of Science, Beijing, China; 11 Ottenby Bird Observatory, BirdLife Sweden, 386 64 Ottenby, Sweden; 12 GLOBE Institute, University of Copenhagen, 1356 Copenhagen, Denmark; 13 School of Ecology, Sun Yat-sen University, Shenzhen, China

**Keywords:** Loop migration, Molt migration, Flight altitude, Geographical barriers, Central-China flyway, Geolocation, Archival GPS, Multi-sensor logger, GeoPressureR

## Abstract

**Background:**

Small songbirds respond and adapt to various geographical barriers during their annual migration. Global flyways reveal the diverse migration strategies in response to different geographical barriers, among which are high-elevation plateaus. However, few studies have been focused on the largest and highest plateau in the world, the Qinghai–Tibet Plateau (QTP) which poses a significant barrier to migratory passerines. The present study explored the annual migration routes and strategies of a population of Siberian Rubythroats (*Calliope calliope*) that breed on the north-eastern edge of the QTP.

**Methods:**

Over the period from 2021 to 2023, we applied light-level geolocators (13 deployed, seven recollected), archival GPS tags (45 deployed, 17 recollected), and CAnMove multi-sensor loggers (with barometer, accelerometer, thermometer, and light sensor, 20 deployed, six recollected) to adult males from the breeding population of Siberian Rubythroat on the QTP. Here we describe the migratory routes and phenology extracted or inferred from the GPS and multi-sensor logger data, and used a combination of accelerometric and barometric data to describe the elevational migration pattern, flight altitude, and flight duration. All light-level geolocators failed to collect suitable data.

**Results:**

Both GPS locations and positions derived from pressure-based inference revealed that during autumn, the migration route detoured from the bee-line between breeding and wintering grounds, leading to a gradual elevational decrease. The spring route was more direct, with more flights over mountainous areas in western China. This different migration route during spring probably reflects a strategy for faster migration, which corresponds with more frequent long nocturnal migration flights and shorter stopovers during spring migration than in autumn. The average flight altitude (1856 ± 781 m above sea level) was correlated with ground elevation but did not differ between the seasons.

**Conclusions:**

Our finding indicates strong, season-dependent impact of the Qinghai–Tibet Plateau on shaping passerine migration strategies. We hereby call for more attention to the unexplored central-China flyway to extend our knowledge on the environment-migration interaction among small passerines.

**Supplementary Information:**

The online version contains supplementary material available at 10.1186/s40462-024-00495-5.

## Introduction

Twice a year, billions of passerine birds migrate along the major flyways in the world [11]. The various topographical, climatic, and biotic patterns, as well as different historical glacial events in different flyways, have selected for a huge diversity of migration strategies [56]. There are certain landscape features in each flyway that are regarded as barriers to migration for all or specific species, e.g., the Mediterranean Sea and the Sahara Desert for the Europe-Africa Flyway [29, 72, 77, 82], the Gulf of Mexico for the Western Atlantic Flyway [9, 79], and the large area of croplands in the central Americas Flyway [24]. These barriers usually consist of a wide range of areas uninhabitable for many species with restricted resources for stopover and refueling [6]. Barriers can also present as a range of high-altitude areas, e.g., the Iran Plateau for the Indo-European Flyway [17, 41, 48], that offer limited resources and more frequent bad weather conditions. These environmental factors may force birds to fly at higher altitudes when migrating across, and to take higher risks of exhaustion and starvation [3]. Consequently, many migratory routes avoid barriers altogether, e.g., by making detours [19, 35, 71], or directly cross them by undertaking uninterrupted flights at high altitude [26, 39, 45, 68].

**Fig. 1:**
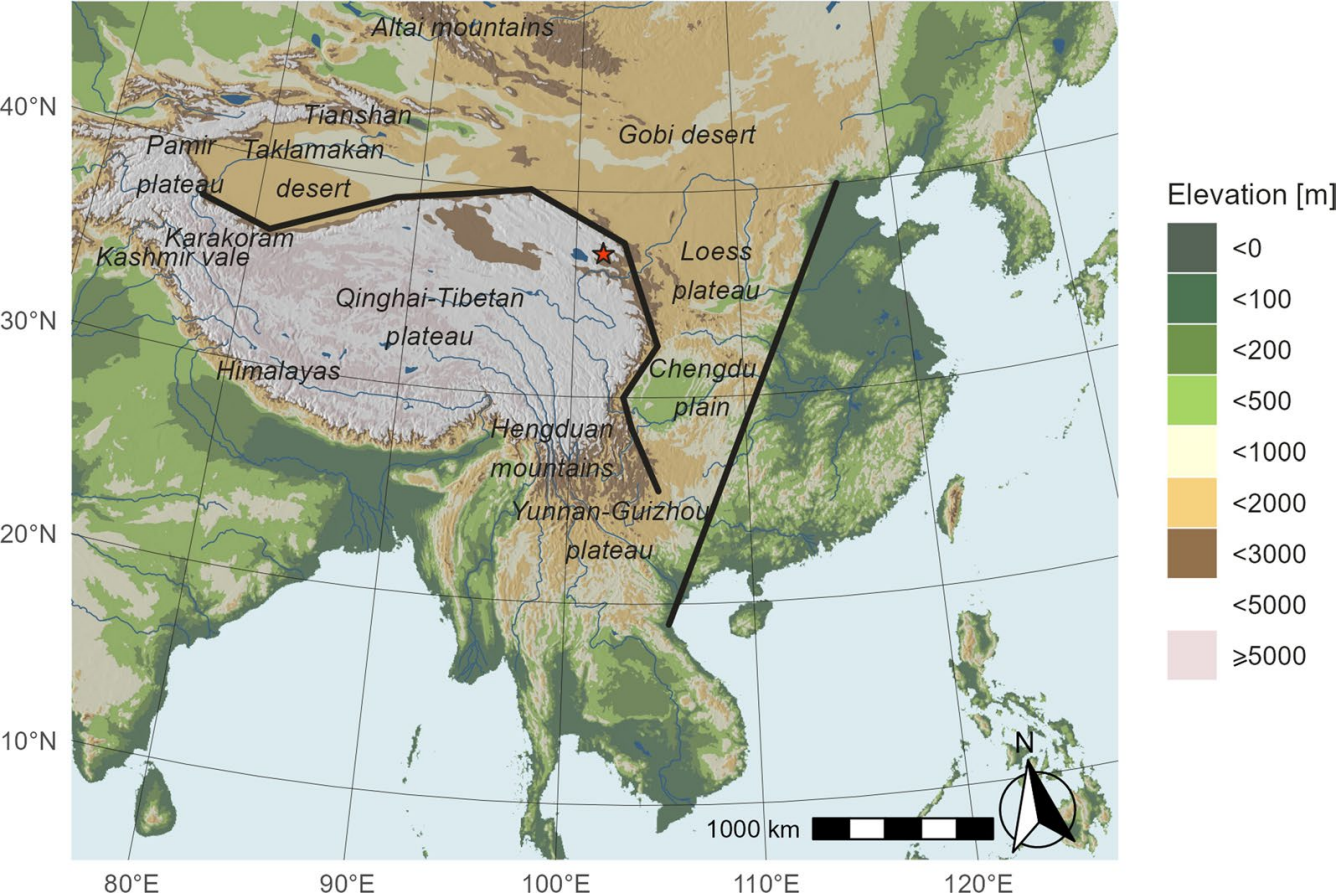
Geography of the study area: black lines indicate the borders of the “Three Stairs of China” landscape pattern. The color palette represents the ground elevation. Major landscape elements that potentially function as geographical barriers to migratory landbirds are highlighted on the map. The red star represents the location of the fieldwork for this project

The Central Asia Flyway (CAF), where the Qinghai–Tibet Plateau (QTP) and adjacent deserts and mountains are regarded as barriers for many migratory passerines, has received much less attention than the European and Americas Flyways [42, 51, 81]. In the range of this flyway, a high diversity of landscapes lies between the Arctic and the Indian Ocean. The elevation profile, apart from the QTP, includes many defined mountain-ranges consisting high peaks reaching 7000 m above sea level (a.s.l.), e.g., the Altai Mountains and the Tianshan Mountains on the northern side, the Hengduan Mountains on the southeastern side, and the southwestern extension of the Himalayas towards Karakoram Mountains, Pamir and Kashmir Range on the western side (Fig. 1. Specifically, the region eastern to the QTP has also been categorized by Chinese geographers as the “second stair of China”. The first stair of China consists of the QTP, with the highest average elevation over 4000 m a.s.l.; the second stair consists of the Mongolian Plateau, the Loess Plateau, the Yunnan–Guizhou Plateau and vast range of mountainous area, with average elevation between 1000 and 2000 m a.s.l.; the third stair consists of the eastern plain area, with the lowest average elevation below 500 m a.s.l. [32].

Given the vast range of geographical challenges along the flyway, it has been hypothesized that passerine migration would be funneled either east or west of the QTP [35]. The area of central to eastern China, which is considered either as part of the CAF, or as part of the East-Asia–Australasia-Flyway (EAAF), and the area around Ladakh and Kashmir, are known to be hotspots for passerine migration [27, 61, 81]. In contrast, very few passerine species are reported to migrate directly across the QTP [26, 43, 61]. Several migratory divides are also reported around the QTP, e.g., Barn Swallows (*Hirundo rustica*) [67, 76], Siberian/Amur Stonechats (*Saxicola maurus*/*stejnegeri*), and Greenish/Two-barred Warblers (*Phylloscopus trochiloides*/*plumbeitarsus*) [33–35]. Unlike larger non-passerine birds, e.g., the Bar-headed Goose (*Anser indicus*) and the Demoiselle Crane (*Grus virgo*) that migrate across the Himalayas with flight altitude over 6000 m a.s.l. [12, 21], the challenge for crossing the barriers may be too much for smaller passerines; thus, they may be more prone to avoid direct confrontation with this major geographical barrier [42].

It is unknown whether the populations of passerine species that breed on the QTP would also avoid geographical barriers during migration. There are many highland-breeding passerine species in the QTP area that are migratory and with wintering grounds south of the QTP, but little do we know about their migration routes. Their adaptation to breeding at high altitudes may offer them the capability to challenge the barriers directly [26, 63], but they may also follow the migration strategy of their lowland breeding relatives.

It has been widely demonstrated that migratory species may have seasonal-different migration strategies. Some species use different routes in autumn and spring, usually referred as “loop migration” [12, 37, 78]. Many species migrate faster in spring than in autumn [40]. Various factors have shown to correlate with the seasonal-different migration strategies, e.g., weather condition, wind direction [21, 54], habitat availability [1, 25], and competition for mating resources in spring [40].

Recent advancement of technology has provided more opportunities for studying the migration of small passerines [71]. Apart from the widely-applied Light-level geolocator (GL) method, the minimized archival GPS technology and the advanced analysis of data from multi-sensor miniature loggers also thrived in the past decade, assisting ornithologists to acquire highly precise migration patterns of small songbirds [8, 49, 50, 52, 64, 68, 69]. In our study, we employed these methods to investigate migration of Siberian Rubythroats (*Calliope calliope*).

The Siberian Rubythroat is a small songbird with lean body mass between 20 and 25 g [18]. Its breeding range spreads from the Ural Mountains to Kamchatka and Anadyr in Russia, northern Mongolia, northern Japan, northern Korea to northeast China, and central China. Rubythroats are known to spend the non-breeding season in coastal areas and islands of East Asia, in South-East Asia, and eastern India [18]. Previous tracking of one of its breeding population from the Russian Far East suggested a rather direct migration route passing through central China towards mainland South-East Asia, thereby largely following the East Asia–Australasia Flyway (EAAF) [27, 28]. However, nothing is known regarding the migration patterns of an isolated breeding population on highlands of central China. This population is sometimes recognized as a separate subspecies *Calliope calliope beicki* [73, 74], but has no known morphological differences compared to other populations [18]. Its breeding range is located in the mountainous area along the transition between the Loess Plateau and the QTP (33–37.5°N, 98–108°E), with an average elevation of around 1500 m a.s.l., The migration route of this population is of special interest as it is located at the crossroads of the pre-defined EAAF and the CAF [42, 81]. In this study, we examined the annual migration patterns of the Siberian Rubythroat population breeding in the central China highland by applying several tracking techniques. We aimed to evaluate how the geographical barriers (e.g., the QTP) are associated with the migration pattern of this species from (1) spatial and (2) temporal perspectives, and (3) to compare the seasonal differences in flight and stopover patterns.

## Methods

### Fieldwork

We carried out our fieldwork in Datong Hui and Tu Autonomous County (simplified as Datong in later contexts), Qinghai, China (37.00–37.04°N, 101.59–101.75°E, 2500 m a.s.l., Figure S1), which is a densely populated region with diverse land-use, e.g., croplands, parks, and villages (Zhao, observational data).

We conducted our fieldwork between 2020 and 2023 in May and June during the early-breeding season, when the male Siberian Rubythroats were intensely displaying and aggressive to playbacks, whereas females remain mostly cryptic and seemingly unresponsive. We used 3- or 6-m-long mist nets in the breeding territories combined with playbacks to catch the breeding adult males. We weighed each caught individual, and equipped the ones weighing at least 21 g with one type of loggers (introduced in the following section) with a double-loop harness method using 1 mm black nylon strings [62]. We also banded the individual with an aluminum ring, provided by the National Bird Banding Centre of China, and an individual-specific combination of color rings. After the handling, we released the bird back to their territories; the same individuals were located and captured by the same method to retrieve the loggers in the following year.

### Tracking devices

We used three types of loggers to track the annual cycle of adult male Siberian Rubythroats: light-level geolocators (GL), archival GPS loggers and multi-sensor loggers developed by the CAnMove lab from Lund University, Sweden (CAnMove logger). The GL is a commonly used miniature tracking device on small birds; it registers the light intensity for geolocation inference [75]. We used the fLight model of GL from Lotek Wireless, Inc. (0.4 g, size 17 mm × 6.5 mm × 3 mm, 7 mm length of the stalk, light registration every 4 min, 9 months of guaranteed battery life). The archival GPS is a slightly heavier data logger than GL; it can provide locations with higher precision but with much shorter battery life. We used the PinPoint-10 archival GPS from Lotek Wireless, Inc. (1.0 g, size 21 mm × 13 mm × 5 mm, 50 mm antenna, rechargeable battery with average allowance of 50 geolocation fixes in 12 months). We manually scheduled the collection of GPS fixes using the life expectancy function in the Lotek interface “PinPoint Host” software (Lotek Wireless Inc.) to maximize battery use and collect as much migration-related data as possible. The schedules for 2020–2021 and 2022–2023 were different: the former was designed to maximize the likelihood that the logger could survive the whole year covering both migration seasons, and the latter was more optimized to the autumn migration window of the focal population (Figure S2). All fixes were set to be collected at 00:00 or 01:00 GMT, which is between 05:00 and 07:00 a.m. for the regions where we expected the birds would be present (GMT + 6 to GMT + 8).

The CAnMove loggers were composed of a light sensor, a barometer, a thermometer, and an accelerometer [7]. The accelerometer measures 6 activity sub-scores evenly in an hour, summed up to an hourly active score (0–30). Each sub-score ranges from 0 to 5, representing the sum of six binary measurements of active/non-active status in a 5-s measuring window. We customized the settings of the logger so we could limit weight of the loggers and maximize the battery life to 12 months (0.85 g, size 20 mm × 10 mm × 2.5 mm, 6 mm light stalk). The tags were set to continuously collect activity data from August 15th, 2021, until the battery dies. The barometer and temperature sensors were programmed to collect pressure and temperature data every hour from August 15th until November 20th, 2021, and from March 15th until May 20th, 2022. The light sensor was scheduled to collect light intensity data every 5 min between December 21st and 26th 2021 to assist in determining the geolocation of the wintering ground.

We deployed 13 GL and 31 archival GPS in 2020 and recollected five GL and 11 archival GPS that collected data successfully in 2021. We deployed 20 CAnMove loggers in 2021 and recollected six that collected data successfully in 2022. However, one out of the six retrieved loggers had a clock drift since January 2022, which disabled the analysis during spring migration. We deployed 12 more archival GPS loggers in 2022, and recollected six in 2023, five of which collected data (Table 1). To evaluate the tag effect, we applied a logistic model to investigate whether logger type and body size (wing length) had any effect on the estimates of return rate.

**Table 1:** Logger deployment and recollection results from the studied breeding population of Siberian Rubythroats

Logger type	Weight	Status	2020	2021	2022	2023	Return rate
GL	0.4 g, < 3% lean body mass	Deployed	13				0.69
		Resighted		9			
		Recollected		5/7^**a**^			
Archival GPS	1.0 g, < 5% lean body mass	Deployed	31		12		0.49
		Resighted		15		6	
		Recollected		11/14^**b**^		5/6^**c**^	
CAnMove logger	0.85 g, < 5% lean body mass	Deployed		20			0.30
		Resighted			6		
		Recollected			6		

^**a**^Out of the seven recollected loggers, two failed to collect data. ^**b**^Out of the fourteen recaptured individuals, three lost their loggers. ^**c**^Out of six recollected loggers, one failed to collect data

We sent the GLs back to Lotek for data extraction. We used the PinPoint Host software to download and process the data from the archival GPS loggers. We sent the CAnMove loggers back to the CAnMove lab for data extraction, and acquired activity, light-level, and pressure and temperature datasets for further analysis.

### Inferring spatial patterns of migration

#### Light-level geolocator data

We followed the standard geolocator data processing manual (geolocationmanual.vogelwarte.ch) and used the “GeoLight” package in R to analyze the GL data [46, 47]. We log-transformed the light data and used the “preprocessLight” function to determine the twilight events with the trial of a set of thresholds. However, due either to dense vegetation habitat that the tagged birds used during the whole non-breeding season, or to molted feathers covering the light stalk, the light-intensity data quality during non-breeding season from all GL data sets was too low for further analysis (Figure S3).

#### Archival GPS data

We used the “HDOP (horizontal dilution of precision)” parameter for each GPS fix as a quality check of accuracy of GPS, and “eRes (Residual error)” parameter as a quality check of accuracy of both the GPS and altitude inference. We maintained the GPS latitude and longitude data if the HDOP < 10; we maintained the altitude data if the eRes > 0.1 (PinPoint Store-on-Board User Manual, Lotek Wireless Inc.). The thresholds were arbitrary and specific to our dataset. As we were using the GPS data solely for route description and visualization, we set a loose threshold to only remove the obviously aberrant datapoints. In total, we filtered out 30/434 datapoints from the dataset with the threshold set-up. We then acquired the ground elevation for all GPS positions (h_g_) in the dataset, and compared it with the altitude registered by the loggers (h_l_). In case of flight events during the geolocation registration, we filtered out those data points if h_l_ – h_g_ > 100 m.

We used the “geosphere” package to calculate the great-circle distance between each geolocation for each individual [31]. Locations recorded within a 300 m range and less than 100 m elevation difference were merged as one stopover site with the average latitude, longitude and elevation. We calculated stopover duration as the number of days between the first and the last date birds were located at the stopover site. We repeated the great-circle distance calculation between consecutive stopover sites, and using the sum of these distances as the total autumn migration distance; we also converted the between-stopover distances into percentages of the total migration distance to represent the progression of migration. Because that the GPS schedules were unevenly spread during the autumn migration in 2020, and the GPS schedules were also different between the 2020 and 2022 dataset; we expected that the calculated migration distance for the 2020 data set might be underestimated due to lack of recording several interim stopovers. We also expected the 2020 estimate to be less precise than the 2022 estimate, due to the lack of information between November 15th and December 14th. However, we believe that the imprecision was negligible as the recorded migration patterns among all loggers were highly similar. We calculated the great-circle distance between the breeding and winter geolocations as the “bee-line” distance. We classified birds to show winter movement if the radius of the winter stationary area was more than 600 m. This value derived from one individual (49994) which differed from the otherwise very small winter home range by moving over 600 m. Our threshold of 600 m thus differentiated this individual from other birds.

#### Atmospheric pressure and activity data

For the subsequent analysis, we defined “stopover” and “rest in between” for different contexts when birds were not flying during the migration periods. Stopover was defined as any non-flying period between two nights of migration flight before reaching the wintering location, regardless of daytime movements. The “rest in between” was defined as a short period (< 6 h) of inactivity within one night when the bird was flying both before and afterwards during the same night.

From the CAnMove logger data, we applied the atmospheric pressure-based geo-positioning method developed by [58, 59] to infer the migration routes from pressure and activity data, following the manual (https://raphaelnussbaumer.com/GeoPressureManual). This approach is based on the R package “GeoPressureR (v2.7)” [60] with three major steps: (1) labeling the pressure and activity timeseries to define flight and stopover events; (2) produce likelihood map of the position of the bird during stopover based on the mismatch of the hourly pressure measurement with a global pressure database (ERA5) [30] (see Nussbaumer et al. [58]), (3) model the trajectory of the bird as a state-space model while accounting for flight duration and wind data (see Nussbaumer et al. [59]). The full description of the method can be found in Additional file 1.

To deal with the potential bias of overestimating flight hours due to the “rest in between” cases resulting in an underestimation of flight speed, we adjusted flight hours to better estimate flight duration. We first calculated the duration of each migration flight event by counting the total flight hours for each event. We then adjusted the duration of active flight during flight events, in hours, by deducting the time with low-intensity activity scores using:

$T_{a} = T_{f} - \frac{1}{6}\sum\limits_{i = 0}^{2} {a_{i} }$, with *T*_*f*_ and *T*_*a*_ referring to the total duration of flight events and the duration of active flight, respectively, and *a*_*i*_ being the contribution of activity score *acc[i]* to *T*_*f*_.

Due to the pre-settings of the CAnMove loggers for maximizing the life-expectancy of the battery, our dataset lacked pressure data between November 20th, 2021, when all birds were still on autumn migration, to March 15th, 2022. Therefore, this period was excluded from further analyses and had no effect on the overall trajectory inference.

After acquiring the inferred migration routes for all individuals, we calculated the cumulative distance for the autumn and spring migration routes. For the autumn migration part that had no pressure data, we assumed a direct path between the last stopover during autumn migration that had pressure data, and the wintering location. We used the great-circle distance for this path to complete the calculation of the total autumn and spring migration distance. Due to the movement model that we used for the migration route inference, short-distance reverse migration or flights that didn’t follow the expected migration direction may be overlooked; therefore, the estimated total migration distance might slightly underestimate the effectively flown distance. We also calculated the great circle distance between the breeding and winter geolocation as the bee-line distance.

We assigned birds to show winter movement if nocturnal activity scores above 5 occurred during the winter periods.

#### Inference of altitudinal movement and migration flight altitude (a.s.l.)

We used the CAnMove logger data to infer the stopover altitude and flight altitude using the empirical equation according to the International Standard Atmosphere model (International Organization for Standardization 1975: ISO 2533:1975):

$$H= -\frac{{T}_{0}}{L} \cdot \left[1-{\left(\frac{P}{{P}_{0}}\right)}^{\frac{1}{5.2561}}\right]$$

where H = altitude (m, a.s.l.), P = pressure from the barometric sensor (hPa or mb), P_0_ = air pressure at sea level (hPa or mb), T_0_ = temperature at sea level (Kelvin) and L = Lapse rate (temperature change per meter increase in altitude, deg/m) [69]. For the equation, we used the standard sea level conditions P_0_ = 1013.25 hPa, T_0_ = 288.15 K (= 15.15 °C) and standard L = − 0.0065 deg m^–1^.

#### Seasonal correlation between flight altitude and stopover elevation

We could not perform statistical analysis on the flight altitude distribution between autumn and spring migration due to the partial lack of pressure data after November 20th, 2021. In addition, the complex landscape and elevation profile in this flyway made it difficult to infer the relative flight height above ground (simplified as relative flight height hereafter) from the distribution of flight altitude. To cope with the problem, we tested the relationship between flight altitude and stopover elevation instead, and inspected whether there were seasonal differences.

We calculated the mean flight altitude with exclusion of the first and the last hour for each flight event as the bird could have been on ascendance or descendance. Therefore, only flight events with duration longer than 2 h were included in this analysis. We also excluded the altitude of the hours labeled as “rest in between”, during which the birds were likely not flying. We defined the stopover elevation before and after each flight event as departure and arrival elevation, calculated from the pressure data at the hour before the flight started and at the hour after the flight ended, respectively. We did not use the mean elevation for each stopover site because the birds often departed from or landed at a slightly different elevation before moving to the stopover site [55]. Hence, the altitudes from the hour right before and after the flight events represent the departure and arrival environment more accurately. We tested the correlation between flight altitude and departure or arrival altitude by season, respectively, using mixed-effects linear regression models. Details of the statistical models are in Additional file 1.

#### Inference of migration phenology

We used both the archival GPS and CAnMove logger data to derive information on the annual phenological patterns. We observed a proportion of tracked individuals having an “early migration” pattern from late July to late August followed by a long stopover for around two months. We assumed that this movement is molt migration based on: (1) adult Siberian Rubythroats’ Summer-Complete (SC) molt pattern after breeding [57], and the observed “early migration” occurrence shortly after breeding, similar to the temporal pattern of molt migration in other species [36], (2) The duration of a SC molt for long-distance migrants is around 7 weeks [38], and the duration of the long stopover after the “early migration” is around 2 months. Therefore, in the archival GPS dataset, we identified the autumn migration departure as the date when the bird made the first long-distance (> 10 km) movement in October, whereas long-distance movements in July and August were defined as molt migration. We could not determine arrival dates on the wintering grounds from some of the archival GPS dataset due to the long interval between scheduled fixes during the relevant period. We identified spring migration departure as the date when the birds made the first long-distance (> 10 km) movements in April, and arrival as the date when they arrived at their breeding locations.

With the CAnMove logger data, we used the activity data to identify the start and the end date of both autumn and spring migration. Similar to the GPS data analysis, we defined the movement in August and early September as molt migration and defined the actual autumn migration start date as the day when the birds carried out the first nocturnal flight (≥ 1 h) in October. We identified the autumn migration arrival date as the day when a bird finished its last long flight (> 3 h). Occasional short winter movements did not obscure this cut-off as they neither occurred within 2 weeks after the arrival date nor lasted longer than 2 h per movement. For spring migration, we identified the start and end date as when the birds carried out the first nocturnal flight in April and when they arrived at their breeding ground after the last nocturnal migration flight.

#### Stopover and flight duration

We extracted the flight and stopover events during both autumn and spring migration to compare seasonal behavioral differences that may be associated with the spatial–temporal migration strategies. We tested whether there are seasonal differences in (1) total duration of stopovers; (2) duration of each stopover event; (3) duration of each flight event per night. We used mixed-effects linear regression models for each test. Details of the statistical models are in Additional file 1.

## Results

### Evaluation of the tag effect

We detected that the birds with CAnMove loggers had a significantly lower return rate than the GL (Estimates = − 1.657, SE = 0.811, t = − 2.043, *p* = 0.045; Additional file 2). The model also suggested a trend that the individuals that did return had longer wings (Estimates = 0.1885, SE = 0.1327, t = 1.421, *p* = 0.160).

### Migration routes and wintering locations

**Fig. 2:**
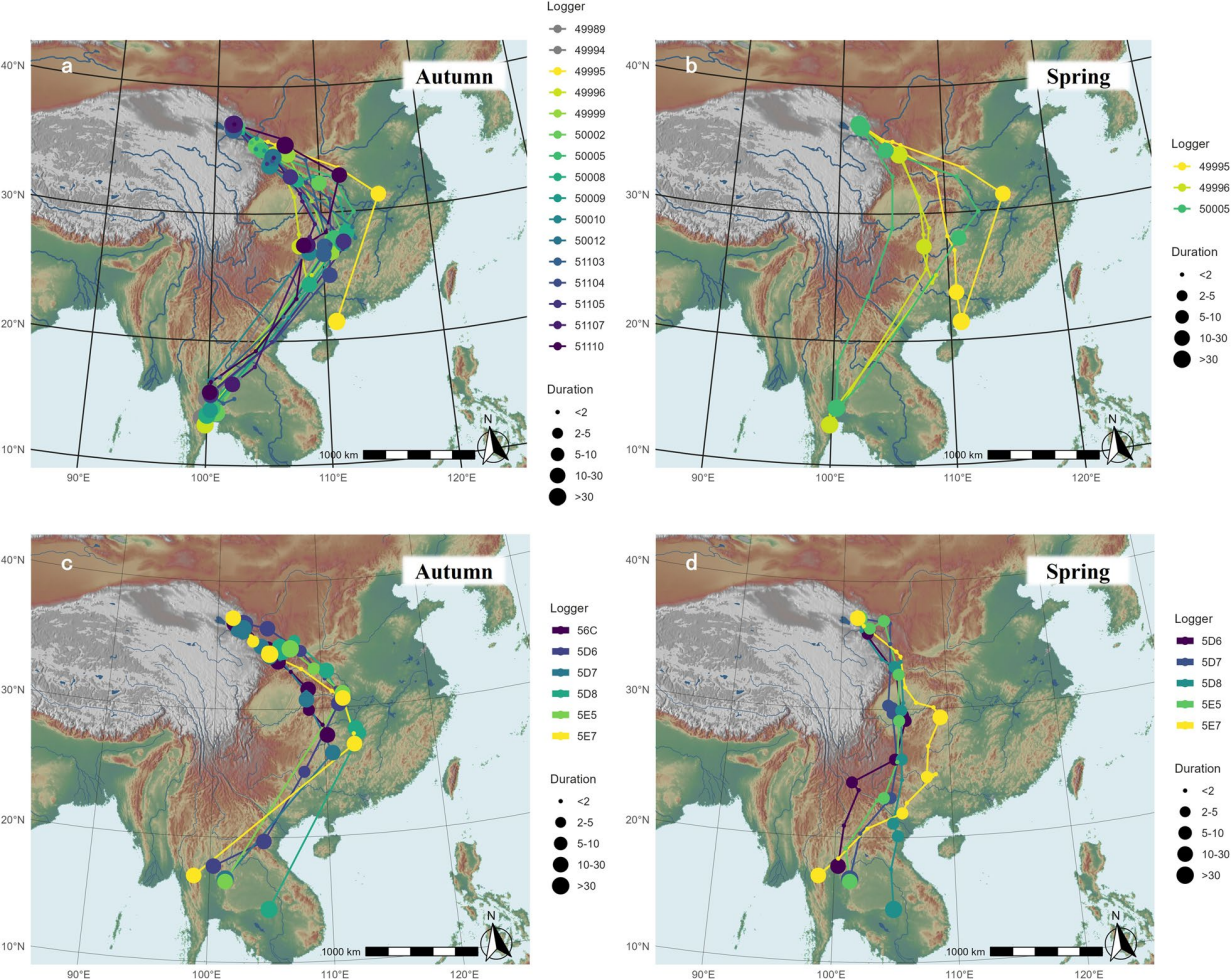
**a–b** Migration routes of Siberian Rubythroats from the Qinghai breeding population based on GPS loggers: **a.** autumn routes (n = 16, note that the route of individual 50012 was not complete, and the route of 50010 was only nearly complete); **b.** whole-year routes (n = 3). **c–d.** Migration routes based on CAnMove loggers: **c.** autumn routes (n = 6); **d.** spring routes (n = 5). Different colors represent different individuals. The size of the dots represents the relative duration of each stopover (in days). Lines represent the bee-line between each position

We acquired 16 autumn tracks from archival GPS logger data (nine complete and two incomplete from 2020, and five complete from 2022) (Fig. 2a–b), and six autumn tracks from CAnMove logger data from 2021 (Fig. 2c–d), all from adult males. All autumn routes started in a SE direction from the breeding site, and most of the individuals switched to SW after arriving in central China in early November before reaching their wintering ground between early December and early January. We located three aggregated long stopover (> 3 days) areas: The first one was in southern Gansu, around 420 km from the breeding site (33.7–36.7°N, 102.6–107.1°E, elevation 1707 ± 452 m a.s.l.; mean ± standard deviation), including the molt sites of 7/13 individuals that conducted molt migration. The second long stopover was close to the range of Qinling Mountains, but on a lower altitude range (30.3–32.9°N, 107.2–111.9°E, elevation 541.9 ± 640.8 m a.s.l.). The third main stopover area was mainly within Hunan and Guizhou province (24.4–28.4°N, 107.9–112.9°E, elevation 473.8 ± 295.2 m a.s.l., Figure S4a).

Most of the tracked individuals wintered in SE Asia, mainly in western Thailand lowland (13.3–17.7°N, 98.7–102.0°E, elevation 42.0 ± 60.3 m a.s.l.) (Figure S4b). This was in line with one local recovery reported in Thailand: a GL-tracked individual from this project ringed June 22nd, 2020 was re-captured in the Bung Boraphet Wildlife Research Station (100.268095°E, 15.702346°N) on January 9th, 2021 by local ringers (Figure S5). One second-year-old individual took a more eastern route in autumn and wintered close to Zhanjiang, China. We documented a few winter movements in 11/20 individuals from both the GPS and CAnMove logger datasets (individual 50010 and 50012 was not included as they didn’t have data from wintering period); the distance was between 0.6 and 46 km from the GPS dataset. Otherwise, the birds mostly remained stationary during the winter.

We acquired three spring routes from the archival GPS data (Fig. 2b), and five spring routes from CAnMove logger data (Fig. 2d). Six of the spring routes followed shorter and straighter paths compared to their autumn routes. Except for the one individual 49995 that wintered in Zhanjiang, China, the spring migration routes crossed the mountainous region in southwest China and passed the Chengdu plain (Fig. 2b, d). The six short routes included long stopovers (> 2 days) within the range of the Chengdu plain (29.1–30.3°N, 104.5–105.7°E, elevation 421.3 ± 108.4 m a.s.l., Figure S4c). The remaining two birds followed different routes, which were almost identical to their autumn routes. All eight spring routes stopped paused close to the first main stopover in autumn in southern Gansu before heading back to the breeding site.

For 14/16 archival GPS-tracked individuals and 5/6 CAnMove logger-tracked individuals that had complete autumn migration tracks, the great-circle distance was 3249 ± 267 km, and the bee-line distance between breeding and wintering site was 2390 ± 164 km. For 3/16 archival GPS-tracked individuals and 5/6 CAnMove logger-tracked individuals that had complete or nearly-complete spring migration tracks, the great-circle distance was 2729 ± 320 km. Overall, the autumn migration distance was 863 ± 194 km longer than the bee-line distance, whereas the spring migration distance was only 398 ± 241 km longer (Table 2).

**Table 2:** Summary of migration distances of the tracked individuals: The total migration distance of Siberian Rubythroats breeding on the QTP during autumn and spring, as well as the great-circle distance between breeding and winter location, and the increased distance (detour) compared to bee-line distance for each tracked individual between 2020 and 2023

Individual (logger name)	Autumn migration distance (km)	Spring migration distance (km)	Bee-line distance^**a**^ (km)	Increased autumn distance^**b**^ (km)	Increased spring distance^**c**^ (km)
49989	3282		2342	940	
49994	3249		2573	676	
49995	2607	2121	1941	666	180
49996	3233	3107	2542	690	564
49999	3142		2359	783	
50002	3370		2525	845	
50005	3513	2665	2494	1018	170
50008	3527		2526	1000	
50009	3320		2506	813	
51103	3584		2406	1179	
51104	3277		2361	917	
51105	3260		2374	886	
51107	2763		2277	486	
51110	3458		2358	1100	
5D6	3060	2602	2164	976	438
5D7	2812	2566	2281	531	285
5D8	3441	2805	2557	883	248
5E5	3109	2705	2294	815	411
5E7	3462	3234	2265	1197	969

^**a**^The great-circle distance between breeding and winter location; ^**b**^The difference between the total migration distance in autumn compared with the bee-line distance; ^**c**^The difference between the total migration distance in spring compared with the bee-line distance

### Elevational movement pattern

**Fig. 3:**
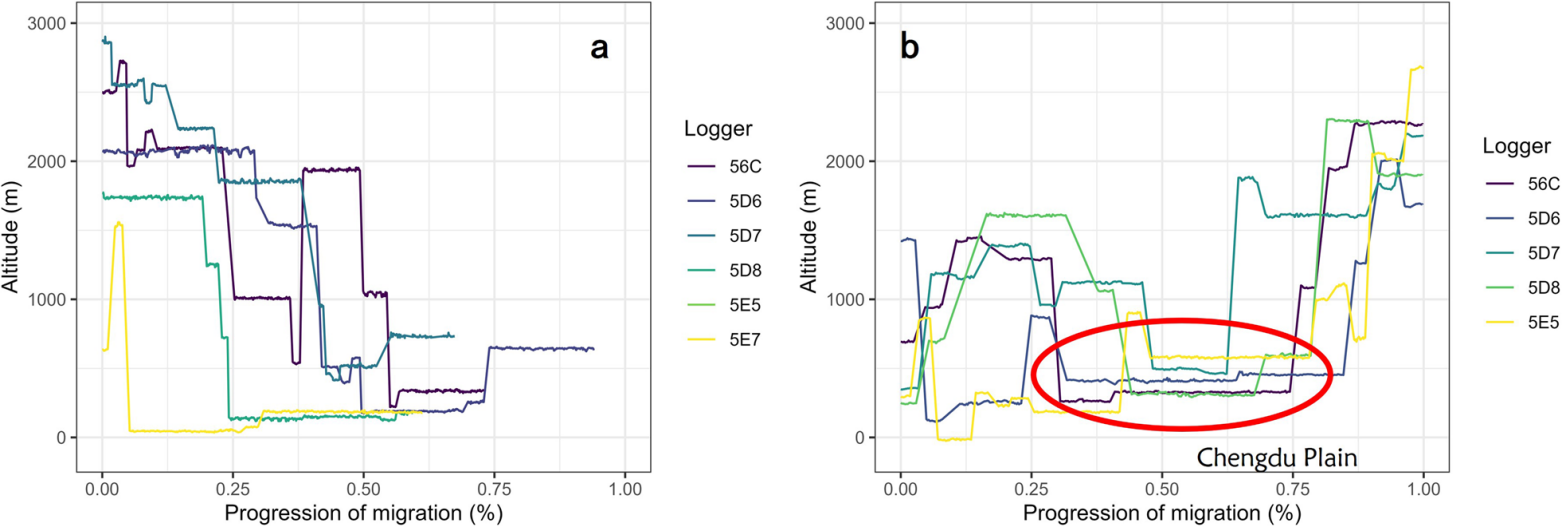
Stopover elevation of Siberian Rubythroats during migration based on CAnMove loggers in **a.** autumn 2021 and **b.** spring 2022. The x-axis represents the proportion of the cumulative duration of the journey against the total duration of the journey. The red circle in panel **b** highlights the passage over the lowland region in the Chengdu Plain. In panel **a**, data in late autumn are lacking because all loggers were set to stop registering pressure data after November 20th, 2021, when none of the birds had finished autumn migration. We assumed a direct migration path between the last stopover during autumn migration that had pressure data and the wintering location; we used the great-circle distance for this path to complete the calculation of the total autumn migration distance

From the archival GPS logger dataset, we documented that the autumn migration has a gradual decrease of ground elevation from up to 2600 m a.s.l. to around 0 m a.s.l. at the wintering sites (Figure S6a). From the CAnMove logger pressure dataset, we acquired more detailed elevational movements in both seasons: In autumn, most of the individuals moved gradually to lower elevation along the stopovers, except for individual 56C, which returned to a stopover at 2000 m a.s.l. after having moved down to a stopover at 1000 m a.s.l. (Fig. 3a, Figure S6b); In spring, most of the individuals had a “rollercoaster” pattern on their altitudinal movements: the altitude of their stopovers first moved up to around 1500 m a.s.l., then down to below 500 m a.s.l., before finally moving up to the breeding altitude at around 2600 m a.s.l. (Fig. 3b, Figure S6c). Exceptionally, individual 5E7 used a similar route during autumn and spring, which unlike the other individuals had no altitude decrease over 1000 m between stopovers (Fig. 3b, Figure S6c).

### Migration flight altitude

**Fig. 4:**
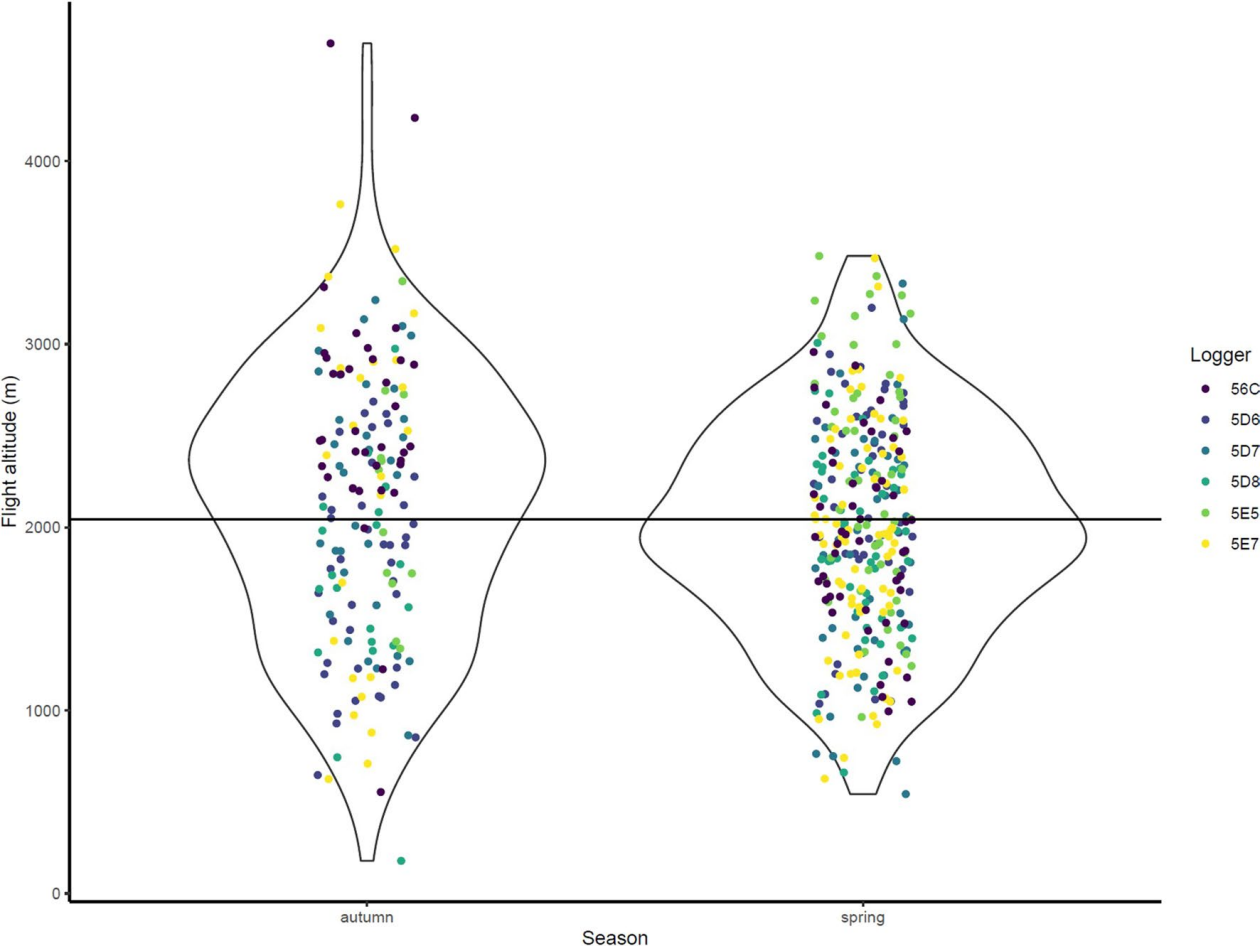
Distribution of mean altitude (a.s.l.) of each flight event of the CAnMove logger-tracked Siberian Rubythroats in 2021–2022 (n = 5). The black line indicates the mean flight altitude during both spring and autumn migration

From the CAnMove logger data, we found that the average flight altitude until November 20th, 2021 was 2119 ± 750 m a.s.l. in autumn (see the Methods section), and 2005 ± 595 m a.s.l. in spring. The highest single-hour flight altitude among all individuals was 4644 m a.s.l. in autumn, and 3482 m a.s.l. in spring (Fig. 4).

### Seasonal correlation between flight altitude and stopover elevation

We observed positive relationships between departure elevation and flight altitude in both autumn and spring. In autumn, the relationship was flight altitude = (0.56 ± 0.13) * departure elevation + (1401 ± 190) (m), R^2^ = 0.30, *p* = 0.0001. In spring, it was flight altitude = (0.44 ± 0.10) * departure elevation + (1585 ± 109) (m), R^2^ = 0.35, *p* < 0.0001 (Figure S7a). We also observed positive relationships between arrival elevation and flight altitude in both autumn and spring. In autumn, the relationship was flight altitude = (0.64 ± 0.11) * arrival elevation + (1452 ± 148) (m), R^2^ = 0.48, *p* < 0.0001. In spring, it was flight altitude = (0.32 ± 0.0867) * arrival elevation + (1612 ± 122) (m), R^2^ = 0.22, *p* = 0.0005 (Figure S7b). Further details can be found in Additional file 2. The slopes for both seasons were similar for departure and flight altitude (df = 76.11, t = − 0.618, *p* = 0.539), whereas for arrival and flight altitude they were significantly lower in spring than in autumn (df = 77.26, t = − 2.161, *p* = 0.0338).

### Phenology

**Fig. 5:**
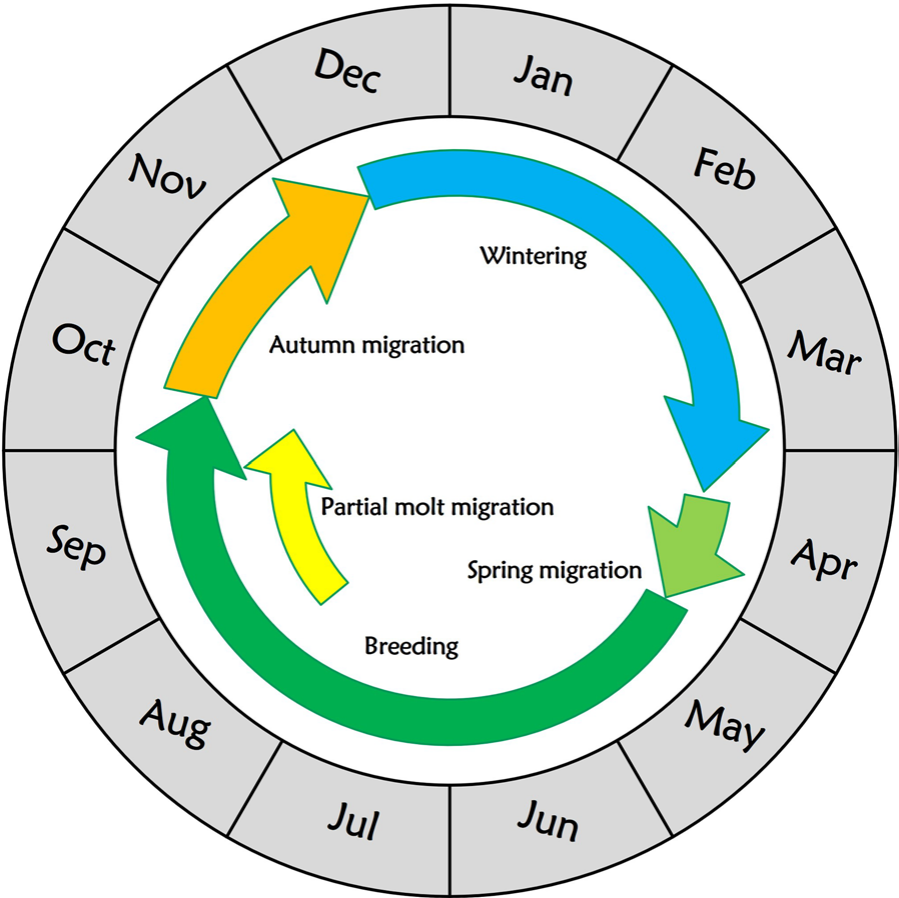
Annual cycle of Siberian Rubythroats breeding on the Qinghai-Tibet Plateau in a calendar

With the combined data set from archival GPS and CAnMove loggers, we acquired 22 autumn (three incomplete) and eight spring datasets regarding migration phenology. The schedules were consistent among three years (Table 3, Fig. 5, Figure S8). In autumn, there were two patterns on the departure time: 13/22 individuals left early from the breeding ground (no later than August 30th) for a molt migration and stayed at a long stopover for around two months before the actual autumn migration; the remaining nine individuals left the breeding ground late: they did not start migration until early to mid-October (Table 3, Figure S8). The arrival date to the wintering ground extracted from both the CAnMove loggers and archival GPS data set from 2023 was between November 23rd and December 28th (Table 3). In spring, all tracked birds in 2021 and 2022 departed from the wintering ground between April 4th and 14th and arrived at the breeding ground between April 24th and May 6th (Table 3).

**Table 3:** Migration phenology and molt migration pattern of tracked Siberian Rubythroats between 2020 and 2023

Individual (logger name)	Molt migration	Molt migration distance^**a**^	Molt migration departure date	Molting stopover duration	Start of autumn migration	End of autumn migration	Duration of autumn migration movement^**b**^	Start of spring migration	End of spring migration	Duration of spring migration movement^**c**^
49989	No				2020-10-08	Before 2020-12-14	39-69 days	NA	NA	NA
49994	Yes	433 km	2020-08-20	67 days	2020-10-20	Before 2020-12-14	26-55 days	Not before 2021-04-05	NA	NA
49995	No				2020-10-11	Before 2021-01-12	64-93 days	2021-04-05	2021-04-30	25 days
49996	Yes	435 km	2020-08-25	56 days	2020-10-20	Before 2020-12-14	26-55 days	Before 2021-04-15	2021-04-30	> 15 days
49999	No				2020-10-20	Before 2020-12-14	26-55 days	NA	NA	NA
50002	Yes	273 km	2020-08-20	64 days	2020-10-17	Before 2021-01-12	58-87 days	NA	NA	NA
50005	Yes	36 km	Before 2020-08-15	> 69 days	2020-10-17	Before 2021-01-12	58-87 days	2021-04-10	NA	~ 20 days
50008	No				2020-10-05	Before 2020-12-14	38-67 days	Not before 2021-04-10	NA	NA
50009	No				2020-10-08	Before 2020-12-14	39-69 days	NA	NA	NA
50010	Yes	293 km	2020-08-20	66 days	2020-10-25	Not before 2021-01-12	50-78 days	NA	NA	NA
50012	Yes	441 km	Before 2020-08-15	> 66 days	2020-10-20	NA	NA	NA	NA	NA
51103	Yes	50 km	Before 2022-08-14	> 59 days	2022-10-26	2022-12-28	56-63 days	NA	NA	NA
51104	No				2022-10-12	2022-12-21	63-70 days	NA	NA	NA
51105	No				2022-10-19	2022-12-21	56-63 days	NA	NA	NA
51107	No				2022-10-12	2022-12-21	63-70 days	NA	NA	NA
51110	Yes	532 km	Before 2022-07-30	> 59 days	2022-10-19	2022-12-28	63-70 days	NA	NA	NA
5D6	Yes	84 km	Before 2021-08-15	> 55 days	2021-10-09	2021-11-23	45 days	2022-04-04	2022-04-24	20 days
5D7	Yes	114 km			2021-10-01	2021-12-13	73 days	2022-04-14	2022-05-04	20 days
5D8	Yes	506 km	Before 2021-08-15	> 70 days	2021-10-11	2021-12-16	66 days	2022-04-14	2022-05-03	19 days
5E5	Yes	555 km	Before 2021-08-15	> 72 days	2021-10-26	2021-12-08	43 days	2022-04-13	2022-05-02	19 days
5E7	Yes	450 km	2021-08-30	56 days	2021-10-28	2021-12-03	36 days	2022-04-08	2022-05-06	28 days
56C	No				2021-10-06	2021-12-05	60 days	2022-04-09	2022-04-26	17 days

^**a**^The great-circle distance between the molting stopover site and the breeding site; ^**b**,**c**^For each individual, the duration of migration movement in autumn or spring accounts for the duration between the first and the last day when a migration flight was conducted. It is not identical to the total duration of migration, as we could not infer the duration of fueling. The estimation of duration of migration movement for archival GPS data retains ambiguity due to the limited data sampling frequency; We estimated the possible range of duration according to the sampling schedule (Figure S1)

### Number and duration of stopovers

In autumn, for the individuals that conducted molt migration in August, the number of stopovers after the resumed migration from October was 11.3 ± 2.1; for the two individuals without molt migration, the number of stopovers were both 17. The only available movement record (individual 5E7) for the molt migration from the breeding ground to the long stopover site had 5 flights and 4 stopovers with duration between 1 and 2 days. In spring, the overall number of stopovers was 10.5 ± 2.4.

**Fig. 6:**
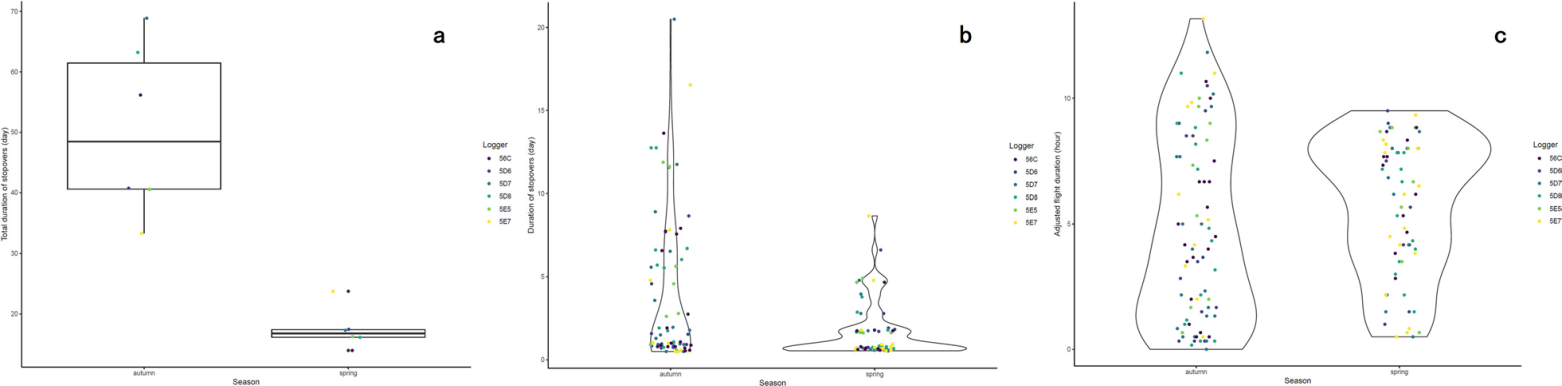
Duration of stopover (**a–b**) and flights (**c**) derived from the data of the CAnMove loggers: **a.** Total duration of stopovers in both seasons. **b.** The distribution of each stopover duration in both seasons; the molting stopovers were not included. **c.** The distribution of each flight duration (calculated from the adjusted flight hours) in both seasons

The total duration of stopovers in autumn (50.5 ± 14.3 days) was significantly longer than in spring (17.5 ± 3.3 days) (df = 10, t = 22.59, *p* < 0.0001, Fig. 6a). The mean duration of stopovers (3.98 ± 4.46 days) in autumn was also significantly longer than in spring (1.66 ± 1.67 days) (df = 137, t = 18.29, *p* < 0.0001, Fig. 6b).

### Duration of migration flight

All individuals migrated exclusively at night in both migration seasons except for one occasion of a 15-h flight by individual 5E7 in autumn that continued into the day (Figure S9a–f). The mean durations of the migration flights were 6.1 ± 3.8 h in autumn and 6.8 ± 2.7 h in spring. The adjusted duration of flight (see the Methods section) was 4.8 ± 3.7 h in autumn and 5.7 ± 2.7 h in spring, and the adjusted autumn flight duration was significantly lower than in spring (df = 149, t = 2.316, *p* = 0.0219, Fig. 6c).

## Discussion

In our study, we presented the annual migration patterns of Siberian Rubythroats breeding on the Qinghai–Tibet Plateau for the first time as observed using different types of miniature data loggers. The migration strategies showed seasonal differences, including the interaction with the impact of landscapes. From the route perspective, the autumn routes were uniformly detoured easternly from the QTP along central China before arriving at their wintering grounds in Thailand, whereas the spring routes were in general shorter and more direct (Fig. 2). From the elevational movement perspective, the autumn elevation was smoothly decreasing along the route, whereas the spring routes passed more high-elevation areas in Yunnan-Guizhou Plateau and the northeast edge of Qinghai–Tibet Plateau (Fig. 3). The flight altitude was on average 2000 m a.s.l. in both seasons. The relative flight height above ground was estimated to be between 1400 and 1600 m when ground elevation is around 0 m a.s.l., and the birds were estimated to migrate more often close to ground when ground elevation is above 2000 m a.s.l., with a few exceptions (Figs. 4, S7). From the phenology perspective, the spring migration was much faster than in autumn, characterized by much shorter duration of stopovers and overall longer flight durations per night (Figs. 5, 6).

### Autumn migration detours from geographical barriers

The combined dataset of GPS tracking and pressure-based geo-positioning demonstrated a clear detoured autumn migration route in our studied population of Siberian Rubythroat. Apart from one bird that took a more eastern route and wintered in the coastal area in Guangdong, China, all the other individuals largely followed the eastern edge of the “second-stair of China”, a continuous mountain chain in central China [32] (Figs. 1, 2a, c). Various environmental factors may play roles in shaping this migration route. First of all, the avoidance of geographical barriers may have resulted in the detour from central China. Compared to the elevation profile along the bee-line between the birds’ breeding and wintering location that have altitudinal barriers up to 5000 m a.s.l., the actual altitudinal decreases along their migration route were more gradual. Another reason for the detour could be related to navigational performance. It has been suggested that geographical cues such as mountain valleys and rivers can function as navigational cues during migration flight (i.e., landlines) [4]. The mountain chain that they are following could therefore be a cue for the decision of changing flight direction in central China. From an evolutionary perspective, the faithfulness towards the wintering ground in SE Asia may also contribute to the shape of the autumn route. The Siberian Rubythroat population breeding in the Russian Far East migrates to non-breeding areas in SE Asia, whereas the breeding population from Hokkaido, Japan, and Kamchatka, Russia, has been shown to spend the non-breeding season in coastal/islandic areas along western Pacific Ocean, as revealed by tracking and ringing data [27, 28]. From a phylogenetic perspective, the population from the Russian Far East is more closely related with the central China population [73]. The fact that none of the individuals from this central China population wintered in the coastal region in eastern China suggests a genetic disposition to migrate to SE Asia for wintering.

### Spring shortcuts along regions with higher elevations

In contrast, the detoured routes were less used in spring migration: 6/8 of the spring routes were straighter than the autumn routes. Apart from a bird that wintered in Zhanjiang (Fig. 2b, d), the spring routes all passed the Yunnan–Guizhou Plateau and Hengduan Mountains in SW China. These complex landscapes and high-elevation profiles (Fig. 1) were not crossed during autumn migrations. The spring routes also crossed the large Chengdu Plain which is surrounded by mountains. Thus, birds needed to pass over mountain regions when entering and departing from the Plain.

Loop-migration patterns are not rare in avian migrants [20, 37, 53, 54, 78]. Classical hypotheses to explain the phenomenon include being urged to return early to the breeding site in order to compete for better territory habitats [56] and seasonal differential weather and wind conditions [5, 6, 21]. We assume that the spring routes employed by our studied individuals are more challenging than the autumn detour routes, possibly exacerbated by higher risks of running out of fuel while migrating across mountainous areas, where the spring has likely not sufficiently advanced to provide abundant food for an easy refuel. We couldn’t detect any obvious wind direction differences between seasons in our analysis (Figures S10, S11), but further modeling may provide more detailed insights on weather and wind effects.

### Faster spring migration is in accordance with the shorter route choice

We found further support for the hypothesis that birds aim to accomplish the migration faster in spring. Even though we couldn’t compare the total duration of autumn and spring migration unbiasedly as we don’t know how long the birds were fueling prior to their first migration flight in each season [3], we noticed that the total duration of stopover was much shorter in spring than in autumn (Fig. 6b). We also discovered that the migration pace in spring was much faster than in autumn: all birds tended to fly longer per night and stay shorter at each stopover in spring. Given that the night length in April is shorter than from October to December in the northern hemisphere, the nighttime was thus more extensively used for migration flight in spring. The birds also tended to spend less time to prepare and refuel before long flights in spring (Figure S12a), suggesting that they fueled more sufficiently at the wintering sites before spring migration than at the breeding or molting sites before autumn migration. As exclusively nocturnal migrants, Siberian Rubythroats stall migration flights during daytime, and the daytime stopover habitat is usually unpredictable. More frequent short stopovers, especially those that were shorter than 24 h, may be associated with fewer chances to refuel, as the birds would lack time to explore during the daytime for proper refuel sites [55].

Nevertheless, the strategy of taking the shorter path in spring associated with higher risks suggests that the benefit must be substantial. The fast journey and earlier arrival can be beneficial to a higher fitness during the breeding phase, e.g., to allow the birds to be more competitive among males and favored by females at the breeding site [40, 65, 66, 70]. In addition, Siberian Rubythroats are highly faithful to their breeding territory ([28], Zhao, personal observational data). An early arrival at the breeding site will allow the birds to occupy their previous territories before it is taken by e.g. first-year breeders. Consequently, it could reduce the energy budget for competition.

Two individuals (49996, 5E7) did not follow a loop-migration pattern in spring, but took a similar route as in autumn (Fig. 2). This choice might offer them easier navigation at the cost of a having to travel longer distances. We only have one phenology dataset of spring migration for this pattern, for which we cannot test statistically whether the detoured spring route lasted longer than the more direct route. The route variation among individuals may reflect their life-history strategies associated with e.g., age and physical condition, which could be tested on a more extensive dataset.

### High migration flight altitude

From a space-use perspective, the lower variation of flight altitude in spring compared to autumn could be correlated with the fact that spring migration is more “roller-coasting” on ground elevation. Given that the routes of Siberian Rubythroats pass numerous mountains that are poorly predictable, it might be more beneficial to stay on a consistently high flight altitude to be capable to deal with barrier-crossings. However, it has also been suggested that it might not be so costly on energy to shift flight altitude drastically [45, 68]. To what extent this seasonal different flight strategy is beneficial still requires more detailed investigations.

Our tracking data suggest that Siberian Rubythroats breeding on the QTP are able to conduct high-altitude flight frequently, which also occur over lowland area regardless of the seasons. The relative flight height above ground in lowland regions (< 500 m a.s.l.) was estimated to be between 1000 and 1400 m (Figure S7). Flight altitude occasionally exceeded 3500 m a.s.l. (Fig. 4), whereas no stopover elevation was above 3000 m a.s.l. (Fig. 3) Tracking studies have recently highlighted the capability of conducting high-altitude migration flight among small passerine species in Euro-Africa flyway in association with barrier-crossing [44, 64, 68]. This consolidates data from field observations and radar studies that have documented frequent flight altitudes of passerines exceeding 1500 m a.s.l. across barriers and in lowland regions, e.g., on the European, American and African continents [10, 16]. Our discoveries on Siberian Rubythroats’ migration flight altitude and height further filled the gap of knowledge along Asian flyways. However, we haven’t been able to differentiate why they fly high along the central China flyway. The preference of a high flight altitude could be associated with tailwind, better weather condition, and clearer visual cues [2]. We would hypothesize that the relative flight height is correlated with landscape or altitude profile along the flight route. However, due to the complexity of landscape along their migration route, neither the pressure-based geo-positioning method nor the archival GPS logger could provide precise landscape or altitude profile between each stopover. Lastly, the recent description of an avivory behavior in a bat species, the Great Evening Bat (*Ia io*) from southern China [23] indicated that there could be a predation risk that forces the birds to fly on a higher altitude even after entering lowland area. There is evidence that Siberian Rubythroat is in this bat’s diet [23], and GPS tracking has suggested that these bats could fly up to 5000 m a.s.l. [22], which could be a result of a predator–prey’s arms race on flight altitude. In our study, we excluded very short-duration migration flights (duration < 2 h) from the relative flight height estimation as we wanted to avoid taking ascendance or descendance event into account. We argue that exclusion of such short flights from our analysis would not lead to a bias: (1) The short flights may not be representative as they could have been disrupted flights from bad weather, or they may not have served for migration purpose; (2) The occurrence rate of short flights (duration < 2 h) was low (9.9%, 15/151); (3) Almost all flight events regardless flight duration were associated with a clear drop of pressure, indicating a obvious rise of altitude (Figure S13).

### The barrier-avoidance migration pattern in a flyway scale

According to the definition of eight major global flyways [11], the migration route of our studied population of Siberian Rubythroat lies between the pre-defined EAAF and the CAF. There hasn’t been any clear definition regarding the boundary between these two flyways, and it has been argued that the eastern QTP flyway could be treated as an independent flyway from either EAAF for CAF [42]. The autumn migration tracks of Siberian Rubythroats from our dataset clearly illustrate an avoidance of the QTP, in contrast to Rubythroats from Russian Far East [27, 28]. This indicates differential selection forces due to the different landscape patterns along the two routes used by the two geographical populations. The same selection force may also influence many other species that migrate between central Siberia and SE Asia, funneling migrants in central China during migration season as a consequence. Alternatively, the “second-stair of China” could also function as a clear navigational cue for migrants using similar routes as our tracked Siberian Rubythroats. We argue that more individual tracking on species breeding close to our studied Siberian Rubythroat populations should be done to guide the delineation of flyways in central-western China.

### The benefits and limits from the new technology

The combination of archival GPS loggers and multi-sensor loggers provides us with enormous opportunities to study passerine migration in detail. For a shrub-dwelling and nocturnally migrating species like Siberian Rubythroat that has little access to sunlight, light-level geolocators couldn’t assemble enough good-quality data for geolocation inference in our dataset. The precision of geolocation inference from light-level geolocator is also not as high as either the GPS data or the pressure-based geolocation data. Furthermore, the accelerometric data can help to infer migration flight activities and daily activities, enabling diverse behavioral research during migration. Our dataset also displayed congruence between the GPS positions and the pressure-based estimations. Based on this, we recommend to use GPS loggers or multi-sensor loggers especially for the many forest-dependent landbirds that move along the Asian flyways.

The consideration of the tag effect needs to be prioritized when designing the tracking research. We observed a potential tag effect on return rate (Table 1, Additional file 2), in that the heavier archival GPS and CAnMove loggers seemed to be associated with a lower return rate than the lighter GL. It is possible that the return rate for CAnMove logger birds was underestimated due to limited searching time due to Covid-19; it is also likely that there was an interannual fluctuation on return rate. Our result is in line with a meta-analysis on carry-over effects of loggers [13]. These authors showed that the weight of the loggers is negatively correlated with the survival rate. Nevertheless, our choice of loggers was strictly following the 5% body weight rule, and the observed return rates for each logger type were all higher than the reported return rate of Siberian Rubythroats from Far East Russia (25%) from Heim et al. [28]. Consequently, we argue that an upper maximal 5% relative weight limit must be strictly followed. We also argue that larger individuals (e.g., with longer wing length) should be prioritized for fitting loggers.

Given that only adult male Siberian Rubythroats have been proved to have strong breeding territory fidelity and with a robust set of strategies for targeted catching over sequential years, we could only deploy and recollect loggers on adult males. Therefore, it was not possible to investigate age or sex differences in migration behavior. Females may exhibit a slower migration speed and later arrival than males in spring [14, 15, 80], which would be a hypothesis to test in future studies when female Rubythroat tracking data becomes available.

In summary, as a basic passerine migration study in a poorly-studied region along a cryptic flyway, we provided novel insights on the migration pattern of this geographical population of Siberian Rubythroats, that may be valuable to disentangle the interaction between geographical barriers and the migration strategies of small passerines in western China. Our results also indicate that central China might be a very important corridor for avian migration in Asia: it could function as a funnel for many northern Asia migrants to travel to their wintering ground in S and SE Asia. Studies that investigate migration behaviors in this region may also be of great value for conservation of avian species. Therefore, we call for more attention on passerines’ migration tracking to improve our understanding of passerine migration in Asia.

### Supplementary Information

Additional file 1: Supplementary methods.

Additional file 2: Reports of statistical analyses.

Supplementary Material 3: Figure S1. The location of fieldwork at Datong, Qinghai.

Supplementary Material 4: Figure S2. The GPS logger schedule for 2020–2021 (left) and 2022–2023 (right), in which the yellow-highlighted dates were scheduled to have a geolocation fix at 00:00 or 01:00 GMT, equivalent to the local time at 5:00 – 7:00 (GMT+6 to +8).

Supplementary Material 5: Figure S3. The light data from the one light-level geolocator (GL484) in 2020–2021. The light-image indicates poor data quality during the non-breeding season of the tracked individual as an example, which applied for all individuals.

Supplementary Material 6: Figure S4. Illustration of the main non-breeding stopovers of Siberian Rubythroats from the studied breeding population: **a**. Autumn long stopovers (> 3days) during migration; different colors represent three categorized stopover ranges, and different shape of points represent different origin of dataset. **b**. Spring long stopovers (> 2days) during migration; different colors represent whether it was the first or second time an individual had long stopovers. **c**. Wintering locations.

Supplementary Material 7: Figure S5. The geolocation of the ringing recovery of one of the GL-tracked individuals (GL485, Ring number C52-8229) in Thailand in January 2021.

Supplementary Material 8: Figure S6. The altitudinal movement of the **a**. archival GPS-tracked Siberian Rubythroats in autumn; **b**–**c**. CAnMove logger-tracked Siberian Rubythroats in both autumn and spring migration; each color represents one individual. 2. **a**. The autumn altitudinal movement of Siberian Rubythroat (n = 10) in 2020; the x-axis “Progression of migration” represents the percentage of cumulative distance that the bird had migrated against its total migration distance; **b**. The autumn elevational movement of Siberian Rubythroats (n = 6) between August 15th to November 20th, 2021. **c**. the spring elevational movement of Siberian Rubythroat (n = 5) between March 20^th^ to May 20^th^, 2022.

Supplementary Material 9: Figure S7. The correlation between flight altitude (m a.s.l.) with **a**. the departure stopover elevation in spring (green) and autumn (red) and **b**. the arrival stopover elevation in spring (green) and autumn (red). A reference line y = x was added as a dashed line in each plot; along and below this line, flight events would be regarded as no/little flight height above ground.

Supplementary Material 10: Figure S8. The cumulative plots of autumn migration of Siberian Rubythroats in **a**. 2020 (n = 10) and **b**. 2022 (n = 5); the y-axis represents the percentage of cumulative distance that the bird had migrated against its total migration distance; each color represents one individual.

Supplementary Material 11: Figure S9. The actograms of all CAnMove logger-tracked Siberian Rubythroats from the Qinghai breeding populations in 2021-2022: **a**–**f** represents actograms from individual 5D6, 5D7, 5D8, 5E5, 5E7, 56C, respectively.

Supplementary Material 12: Figure S10. The migration flight speed and wind speed of each flight event, estimated from the CAnMove logger dataset. The “gs” stands for ground speed, the “as” stands for air speed, and the “ws” stands for wind speed.

Supplementary Material 13: Figure S11. The inferred migration routes and the inferred wind direction during each flight event. The arrows on migration paths represent the wind directions during the flight event. Lighter-colored arrows indicate greater alignment of the flight and wind direction during the flight event.

Supplementary Material 14: Figure S12. The correlation between the flight duration and the stopover duration **a**. before or **b**. after each flight event (adjusted flight hours); red dots and line represents dataset from autumn, and green dots and line represents dataset from spring.

Supplementary Material 15: Figure S13. The raw annual pressure data set from each CAnMove logger. The grey dash-line between November 20^th^, 2021 and March 15^th^, 2022 represents the lack of pressure data due to the data-collection settings.

## Data Availability

The archival GPS data can be accessed on Movebank as a repository: 10.5441/001/1.333. The CAnMove logger data can be accessed on github repository 10.5281/zenodo.10490629. The Light-level geolocator data can be requested from the corresponding author (T.Z.).
