# Supplementary material for: Seasonal migration patterns of Siberian Rubythroat (Calliope calliope) facing the Qinghai–Tibet Plateau
Source: Mov Ecol. 2024 Aug 1;12:54. doi: 10.1186/s40462-024-00495-5 (PMC11295652; doi:10.1186/s40462-024-00495-5)
Supplement: Supplementary file 1 — Additional file 1: Supplementary methods. [file 40462_2024_495_MOESM1_ESM.docx]

# Additional file 1

**Part 1:** Detailed process and settings for the pressure-based geo-positioning

The working pipeline is based on the R package GeoPressureR v2.7. Here we briefly summarized the method, and the detailed manual can be found at https://raphaelnussbaumer.com/GeoPressureManual/.

We first manually identified flight events based on pressure and activity data following the GeoPressureManual recommendation (https://raphaelnussbaumer.com/GeoPressureManual/labelling-tracks.html [10.5281/zenodo.10799355](https://zenodo.org/doi/10.5281/zenodo.10799355)). We initiated the label automatically following criteria: datapoints were labelled as “flight” if they had intense activity (acc[2–5]≠0) during local night time that may also have been associated with drop of pressure value. If the intense activity was not continuous during one night (i.e. acc[2–5] = 0 during some hours) we labelled these inactive hours as “rest in between”. We then manually adjusted these labels with TRAINSET (trainset.geocene.com), which allowed us to visually explore activity scores and air pressure measurements of events in parallel. We also labeled abnormal pressure points to exclude them from further analysis. The flight labeled are used to determined flight duration and stationary periods corresponding to periods where the bird’s position is assumed to be constant.

Secondly, we estimate the position at each stationary periods based on pressure data (Nussbaumer, 2023a). See <https://raphaelnussbaumer.com/GeoPressureManual/pressure-map.html> for further detail on this procedure. The pressure measurement is essentially compared to the ERA-5 pressure database to determine position on the map matching these measurements. See function (geopressure_map() <https://raphaelnussbaumer.com/GeoPressureR/reference/geopressure_map.html>). This step results in a probability map of position for each stationary periods.

Thirdly, we inferred the trajectory by combining the pressure maps (see above), windspeed data and airspeed distribution function in the model developed for multi-sensor geolocator (Nussbaumer, 2023b), (See <https://raphaelnussbaumer.com/GeoPressureManual/trajectory-with-wind.html> for further details of this procedure). We downloaded the wind data (U and V) from the ERA5 database for each flight ( use the function tag_download_wind() ), accounting for the position and altitude of the bird during the flight. The model uses a graphical approach to compute the probability of all possible positions and transitions of the birds. From this model, we extract the most likely path using the function graph_most_likely() (<https://raphaelnussbaumer.com/GeoPressureR/reference/graph_most_likely.html>)

The full analysis details can be found in the github repository: <https://doi.org/10.5281/zenodo.10490629>

**Part 2:** Detailed description of the statistic models

**Seasonal correlation between flight altitude and stopover elevation**

we tested the correlation between flight altitude and stopover elevation and whether there were seasonal differences. We estimated how mean flight altitude varied with season and **departure altitude** using a mixed-effects linear regression model with normal error distribution.

The fixed effects of the model took the form

$h_{flight} \sim N\left( \mu_{h}, \sigma_{h} \right)$ with

$\mu_{h}=\alpha_{autumn}+ \beta_{spring}\times x_{1}+\beta_{departure}\times x_{2}$,

where $h_{flight}$ is the flight altitude with a normal error distribution with mean $\mu_{h}$, which we estimated using the migratory season of the observation, with the effect for autumn migration $\beta_{autumn}$ representing the intercept term, and the departure altitude as predictors. We further included the unique identifier of the logger as a random effect term in the model, and explored whether the effect of departure altitude had an interactive effect with season. This was not the case, and the interaction term was not included in the final model. We used the intercept estimation from the model as one estimation of relative flight height for each season.

We also considered the alternative model using season and **arrival altitude** as predictors, using

$h_{flight} \sim N\left( \mu_{h}, \sigma_{h} \right)$ with

$\mu_{h}=\alpha_{autumn}+ {(\beta}_{arrival,autumn}+\beta_{autumn})\times x_{1}+{(\beta}_{arrival, spring}+\beta_{spring})\times x_{2}$ ,

We found that for this model, however, that the interaction term between season and arrival altitude was significant, and so it was retained in the model. We again included the unique identifier of the logger as a random effect term in the model.

As the interaction between season and arrival was significant, yet the random factor was not; we split the dataset by season and ran separate models for each season: lmer(altitude ~ arrival). We used the intercept estimation from the model as another estimation of relative flight height for each season.

**Stopover and flight duration**

We extracted the flight and stopover events during both autumn and spring migration to compare the seasonal behavioral difference that may be associated with the spatial-temporal migration strategies.

We square-rooted transformed stopover duration (hours) for both of the datasets to normalize the distribution of the data; we then estimated mean stopover duration, and how it varied with season using a mixed-effects linear regression model, using

$d_{s}\sim N\left( \mu_{d}, \sigma_{d} \right)$ with

$\mu_{d}=\alpha_{autumn}+ \beta_{spring}\times x_{1}$,

to estimate the mean stopover duration $\mu_{d}$ for autumn (corresponding to the intercept) and spring migration, respectively. We assumed that the model error could be adequately described using a normal error distribution, and included logger identifier as a random effect term, representing individuals. We applied the same model to both the short and long stopover duration dataset.

We also tested whether there is seasonal difference for the flight duration. We square-rooted transformed flight duration (hours) for both of the datasets to normalize the distribution of the data; we then estimated mean flight duration, and how it varied with season using a mixed-effects linear regression model, using

$d_{s}\sim N\left( \mu_{d}, \sigma_{d} \right)$ with

$\mu_{d}=\alpha_{autumn}+ \beta_{spring}\times x_{1}$,

to estimate the mean flight duration $\mu_{d}$ for autumn (corresponding to the intercept) and spring migration, respectively. We assumed that the model error could be adequately described using a normal error distribution, and included logger identifier as a random effect term, representing individuals. We used the unprocessed flight duration from the raw dataset, as well the flight duration using the adjusted hours for running the model.
