# Supplementary material for: Seasonal migration patterns of Siberian Rubythroat (Calliope calliope) facing the Qinghai–Tibet Plateau
Source: Mov Ecol. 2024 Aug 1;12:54. doi: 10.1186/s40462-024-00495-5 (PMC11295652; doi:10.1186/s40462-024-00495-5)
Supplement: Supplementary file 2 — Additional file 2: Reports of statistical analyses. [file 40462_2024_495_MOESM2_ESM.docx]

Additional file 2 Statistical reports included in the study

| 1-i | **Correlation between flight altitude and departure elevation** | | | | | |  | | | |  | |  | |
| --- | --- | --- | --- | --- | --- | --- | --- | --- | --- | --- | --- | --- | --- | --- |
|  | Formula: altitude ~ season * departure + (1 \| logger) | | | | | |  | | | |  | |  | |
|  | *Random effects:* |  | |  | |  |  | | | |  | |  | |
|  | **Groups** | **Name** | | **Variance** | | **Std.Dev.** |  | | | |  | |  | |
|  | logger | (Intercept) | | 55929 | | 236.5 |  | | | |  | |  | |
|  | Residual |  | | 235057 | | 484.8 |  | | | |  | |  | |
|  | Number of obs: 82, groups: logger, 6 | | | | | |  | | | |  | |  | |
|  | *Fixed effects:* |  | |  | |  |  | | | |  | |  | |
|  |  | **Estimate** | | **Std. Error** | | **df** | **t value** | | | | **Pr(>\|t\|)** | |  | |
|  | (Intercept) | 1402.58541 | | 181.48412 | | 25.19404 | 7.728 | | | | 4.16E-08 | | *** | |
|  | seasonspring | 253.41544 | | 197.92586 | | 75.62411 | 1.28 | | | | 2.04E-01 | |  | |
|  | departure | 0.53899 | | 0.10531 | | 77.32284 | 5.118 | | | | 2.19E-06 | | *** | |
|  | seasonspring:departure | -0.09313 | | 0.1508 | | 76.11153 | -0.618 | | | | 5.39E-01 | |  | |
| 1-ii | **Correlation between flight altitude and departure elevation in autumn** | | | | | | | | | |  | |  | |
|  | Formula: altitude ~ departure, data = autumn | | | | |  |  | | | |  | |  | |
|  |  | **Estimate** | | **Std. Error** | |  | **t value** | | | | **Pr(>\|t\|)** | |  | |
|  | (Intercept) | 1401.3738 | | 190.3051 | |  | 7.364 | | | | 2.25E-08 | | *** | |
|  | departure | 0.5554 | | 0.1271 | |  | 4.37 | | | | 1.23E-04 | | *** | |
|  | Residual standard error: 620.9 on 32 degrees of freedom | | | | |  |  | | | |  | |  | |
|  | Multiple R-squared: 0.3737, | Adjusted R-squared: 0.3541 | | | | |  | | | |  | |  | |
|  | F-statistic: 19.09 on 1 and 32 DF, p-value: 0.0001227 | | | | |  |  | | | |  | |  | |
| 1-iii | **Correlation between flight altitude and departure elevation in spring** | | | | | |  | | | |  | |  | |
|  | Formula: altitude ~ departure, data = spring | | | | |  |  | | | |  | |  | |
|  |  | **Estimate** | | **Std. Error** | |  | **t value** | | | | **Pr(>\|t\|)** | |  | |
|  | (Intercept) | 1.59E+03 | | 1.09E+02 | |  | 14.528 | | | | <2e-16 | | *** | |
|  | departure | 4.42E-01 | | 9.63E-02 | |  | 4.594 | | | | 3.39E-05 | | *** | |
|  | Residual standard error: 441.9 on 46 degrees of freedom | | | | |  |  | | | |  | |  | |
|  | Multiple R-squared: 0.3145, | Adjusted R-squared: 0.2996 | | | | |  | | | |  | |  | |
|  | F-statistic: 21.1 on 1 and 46 DF, p-value: 3.388e-05 | | | | |  |  | | | |  | |  | |
| 2-i | **Correlation between flight altitude and arrival elevation** | | | | | |  | | | |  | |  | |
|  | Formula: altitude ~ season * arrival + (1 \| logger) | | | | | |  | | | |  | |  | |
|  | *Random effects:* |  | |  | |  |  | | | |  | |  | |
|  | **Groups** | **Name** | | **Variance** | | **Std.Dev.** |  | | | |  | |  | |
|  | logger | (Intercept) | | 41299 | | 203.2 |  | | | |  | |  | |
|  | Residual |  | | 226216 | | 475.6 |  | | | |  | |  | |
|  | Number of obs: 82, groups: logger, 6 | | | | | |  | | | |  | |  | |
|  | *Fixed effects:* |  | |  | |  |  | | | |  | |  | |
|  |  | **Estimate** | | **Std. Error** | | **df** | **t value** | | | | **Pr(>\|t\|)** | |  | |
|  | (Intercept) | 1461.5725 | | 154.2681 | | 21.6909 | 9.474 | | | | 3.65E-09 | | *** | |
|  | seasonspring | 199.4029 | | 182.4047 | | 75.7941 | 1.093 | | | | 2.78E-01 | |  | |
|  | arrival | 0.6258 | | 0.1037 | | 77.9983 | 6.033 | | | | 5.07E-08 | | *** | |
|  | seasonspring:arrival | -0.2963 | | 0.1371 | | 77.2574 | -2.161 | | | | 3.38E-02 | | * | |
| 2-ii | **Correlation between flight altitude and arrival elevation in autumn** | | | | | |  | | | |  | |  | |
|  | Formula: altitude ~ arrival, data = autumn | | |  | |  |  | | | |  | |  | |
|  |  | **Estimate** | | **Std. Error** | |  | **t value** | | | | **Pr(>\|t\|)** | |  | |
|  | (Intercept) | 1452.4977 | | 147.992 | |  | 9.815 | | | | 3.58E-11 | | *** | |
|  | arrival | 0.6411 | | 0.1137 | |  | 5.636 | | | | 3.12E-06 | | *** | |
|  | Residual standard error: 555.8 on 32 degrees of freedom | | | | |  |  | | | |  | |  | |
|  | Multiple R-squared: 0.4982, | Adjusted R-squared: 0.4825 | | | | |  | | | |  | |  | |
|  | F-statistic: 31.77 on 1 and 32 DF, p-value: 3.119e-06 | | | | |  |  | | | |  | |  | |
| 2-iii | **Correlation between flight altitude and arrival elevation in spring** | | | | | |  | | | |  | |  | |
|  | Formula: altitude ~ arrival, data = spring | | |  | |  |  | | | |  | |  | |
|  |  | **Estimate** | | **Std. Error** | |  | **t value** | | | | **Pr(>\|t\|)** | |  | |
|  | (Intercept) | 1.61E+03 | | 1.22E+02 | |  | 13.227 | | | | <2e-16 | | *** | |
|  | arrival | 3.24E-01 | | 8.67E-02 | |  | 3.738 | | | | 5.13E-04 | | *** | |
| 3-i | **Seasonal comparison of total stopover duration** | | | | |  |  | | | |  | |  | |
|  | Formula: sq.sa ~ season + (1 \| logger) | | |  | |  |  | | | |  | |  | |
|  | *Random effects:* |  | |  | |  |  | | | |  | |  | |
|  | **Groups** | **Name** | | **Variance** | | **Std.Dev.** |  | | | |  | |  | |
|  | logger | (Intercept) | | 0 | | 0 |  | | | |  | |  | |
|  | Residual |  | | 14 | | 3.742 |  | | | |  | |  | |
|  | Number of obs: 12, groups: logger, 6 | | |  | |  |  | | | |  | |  | |
|  | *Fixed effects:* |  | |  | |  |  | | | |  | |  | |
|  |  | **Estimate** | | **Std. Error** | | **df** | **t value** | | | | **Pr(>\|t\|)** | |  | |
|  | (Intercept) | 34.512 | | 1.528 | | 10 | 22.592 | | | | 6.50E-10 | | *** | |
|  | seasonspring | -14.105 | | 2.16 | | 10 | -6.529 | | | | 6.65E-05 | | *** | |
| 3-ii | **Seasonal comparison of stopover duration** | | | | |  |  | | | |  | |  | |
|  | Formula: sq.sa ~ season + (1 \| logger) | | |  | |  |  | | | |  | |  | |
|  | *Random effects:* |  | |  | |  |  | | | |  | |  | |
|  | **Groups** | **Name** | | **Variance** | | **Std.Dev.** |  | | | |  | |  | |
|  | logger | (Intercept) | | 0 | | 0 |  | | | |  | |  | |
|  | Residual |  | | 0.6873 | | 0.829 |  | | | |  | |  | |
|  | Number of obs: 139, groups: logger, 6 | | |  | |  |  | | | |  | |  | |
|  | *Fixed effects:* |  | |  | |  |  | | | |  | |  | |
|  |  | **Estimate** | | **Std. Error** | | **df** | **t value** | | | | **Pr(>\|t\|)** | |  | |
|  | (Intercept) | 1.724 | | 0.0951 | | 137 | 18.129 | | | | 2.00E-16 | | *** | |
|  | seasonspring | -0.5446 | | 0.1412 | | 137 | -3.855 | | | | 1.77E-04 | | *** | |
| 4-i | **Seasonal comparison of flight duration** | | |  | |  |  | | | |  | |  | |
|  | Formula: sq.hr ~ season + (1 \| logger) | | |  | |  |  | | | |  | |  | |
|  | *Random effects:* |  | |  | |  |  | | | |  | |  | |
|  | **Groups** | **Name** | | **Variance** | | **Std.Dev.** |  | | | |  | |  | |
|  | logger | (Intercept) | | 0 | | 0 |  | | | |  | |  | |
|  | Residual |  | | 0.6962 | | 0.8344 |  | | | |  | |  | |
|  | *Fixed effects:* |  | |  | |  |  | | | |  | |  | |
|  |  | **Estimate** | | **Std. Error** | | **df** | **t value** | | | | **Pr(>\|t\|)** | |  | |
|  | (Intercept) | 1.98627 | | 0.09214 | | 149 | 21.557 | | | | <2e-16 | | *** | |
|  | seasonspring | 0.31569 | | 0.13631 | | 149 | 2.316 | | | | 2.19E-02 | | * | |
| 4-ii | **Correlation between flight duration and departure stopover duration** | | | | | |  | | | |  | |  | |
|  | formula = adj.hr ~ last.st + season | | |  | |  |  | | | |  | |  | |
|  |  | **Estimate** | | **Std. Error** | |  | **t value** | | | | **Pr(>\|t\|)** | |  | |
|  | (Intercept) | 3.88382 | | 0.48443 | |  | 8.017 | | | | 4.41E-13 | | *** | |
|  | last.st | 0.25139 | | 0.07642 | |  | 3.29 | | | | 0.00128 | | ** | |
|  | seasonspring | 1.10203 | | 0.56201 | |  | 1.961 | | | | 0.05193 | | . | |
|  | Residual standard error: 3.133 on 136 degrees of freedom | | | | | |  | | | |  | |  | |
|  | Multiple R-squared: 0.07975, | Adjusted R-squared: 0.06622 | | | | |  | | | |  | |  | |
|  | F-statistic: 5.893 on 2 and 136 DF, p-value: 0.003512 | | | | |  |  | | | |  | |  | |
| 4-iii | **Correlation between flight duration and arrival stopover duration in autumn** | | | | | | | | | |  | |  | |
|  | formula = adj.hr ~ next.st, data = autumn | | |  | |  |  | | | |  | |  | |
|  |  | **Estimate** | | **Std. Error** | |  | **t value** | | | | **Pr(>\|t\|)** | |  | |
|  | (Intercept) | 5.55564 | | 0.57953 | |  | 9.586 | | | | 1.15E-14 | | *** | |
|  | next.st | -0.10822 | | 0.09237 | |  | -1.172 | | | | 0.245 | |  | |
|  | Residual standard error: 3.664 on 75 degrees of freedom | | | | |  |  | | | |  | |  | |
|  | Multiple R-squared: 0.01797, | Adjusted R-squared: 0.00488 | | | | |  | | | |  | |  | |
|  | F-statistic: 1.373 on 1 and 75 DF, p-value: 0.2451 | | | | |  |  | | | |  | |  | |
| 4-iv | **Correlation between flight duration and arrival stopover duration in spring** | | | | | | | | |  | |  | |  |
|  | formula = adj.hr ~ next.st, data = autumn | | | | | | |  |  |  | |  | |  |
|  |  | | **Estimate** | | **Std. Error** | | |  | **t value** | **Pr(>\|t\|)** | |  | |  |
|  | (Intercept) | | 7.5454 | | 0.4716 | | |  | 16.001 | <2e-16 | | *** | |  |
|  | next.st | | -0.8674 | | 0.1855 | | |  | -4.675 | 1.67E-05 | | *** | |  |
|  | Residual standard error: 2.397 on 61 degrees of freedom | | | | | | | |  |  | |  | |  |
|  | Multiple R-squared: 0.2638, | | Adjusted R-squared: 0.2517 | | | | | |  |  | |  | |  |
|  | F-statistic: 21.86 on 1 and 61 DF, p-value: 1.67e-05 | | | | | | |  |  |  | |  | |  |
| 5 | **Evaluation of the tag effect** | |  | |  | | |  |  |  | |  | |  |
|  | formula = return ~ Wing + LoggerType, family = binomial | | | | | | | |  |  | |  | |  |
|  |  | | **Estimate** | | **Std. Error** | | |  | **t value** | **Pr(>\|t\|)** | |  | |  |
|  | (Intercept) | | -13.8077 | | 10.2852 | | |  | -1.342 | 0.184 | |  | |  |
|  | Wing | | 0.1885 | | 0.1327 | | |  | 1.421 | 0.16 | |  | |  |
|  | LoggerTypeGPS | | -0.6845 | | 0.7155 | | |  | -0.957 | 0.342 | |  | |  |
|  | LoggerTypeMUL | | -1.6575 | | 0.8115 | | |  | -2.043 | 0.045 | | * | |  |
|  | Residual standard error: 1.027 on 68 degrees of freedom | | | | | | | |  |  | |  | |  |
|  | Multiple R-squared: 0.004772, Adjusted R-squared: -0.03914 | | | | | | | |  |  | |  | |  |
|  | F-statistic: 0.1087 on 3 and 68 DF, p-value: 0.9547 | | | | | | |  |  |  | |  | |  |
|  | *MUL represents CAnMove logger | | | |  | | |  |  |  | |  | |  |
