# Supplementary figures and images for: Seasonal migration patterns of Siberian Rubythroat (Calliope calliope) facing the Qinghai–Tibet Plateau
Source: Mov Ecol. 2024 Aug 1;12:54. doi: 10.1186/s40462-024-00495-5 (PMC11295652; doi:10.1186/s40462-024-00495-5)

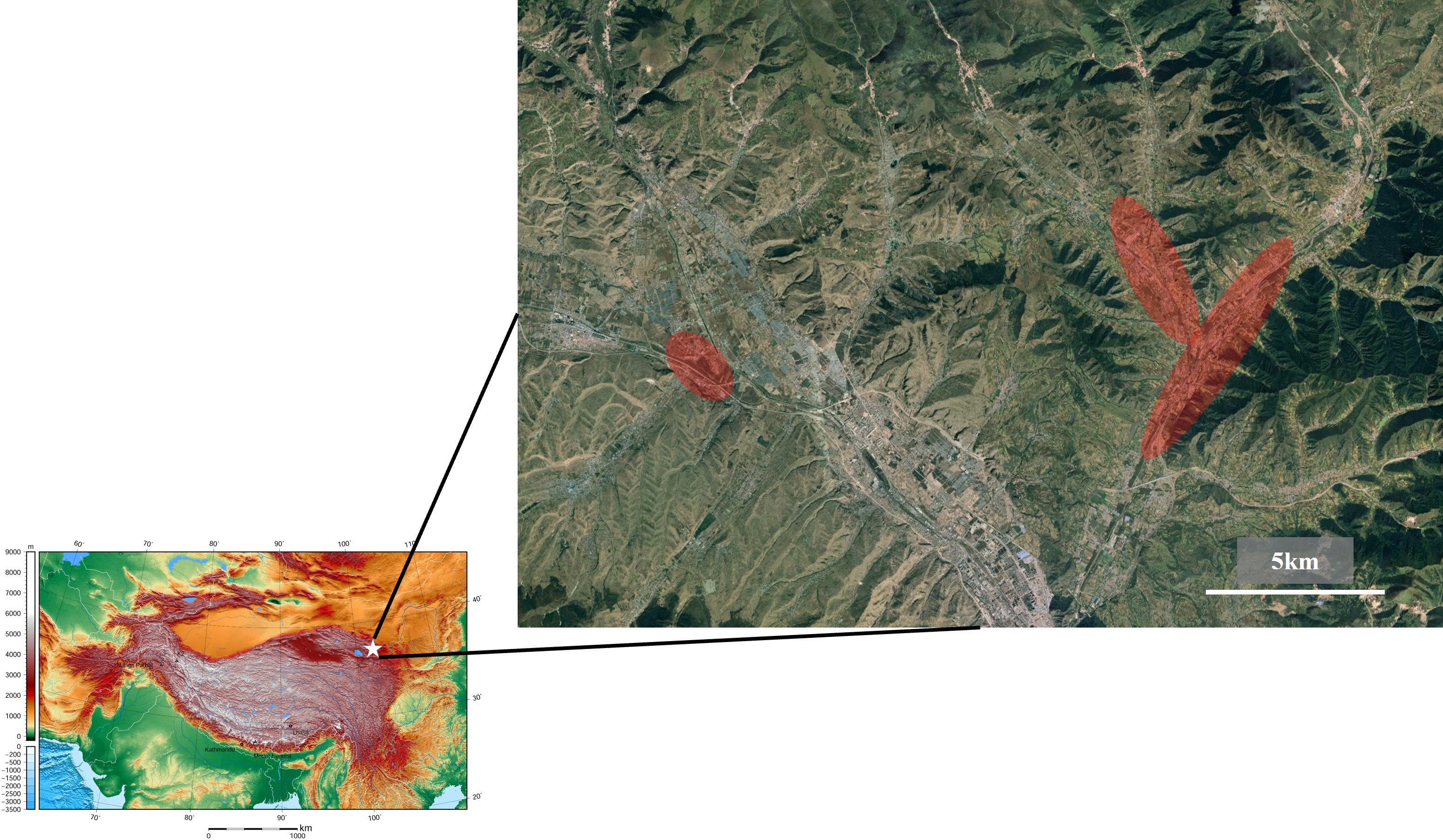

Supplement: Supplementary file 3 — Supplementary Material 3: Figure S1. The location of fieldwork at Datong, Qinghai. [file 40462_2024_495_MOESM3_ESM.jpg]

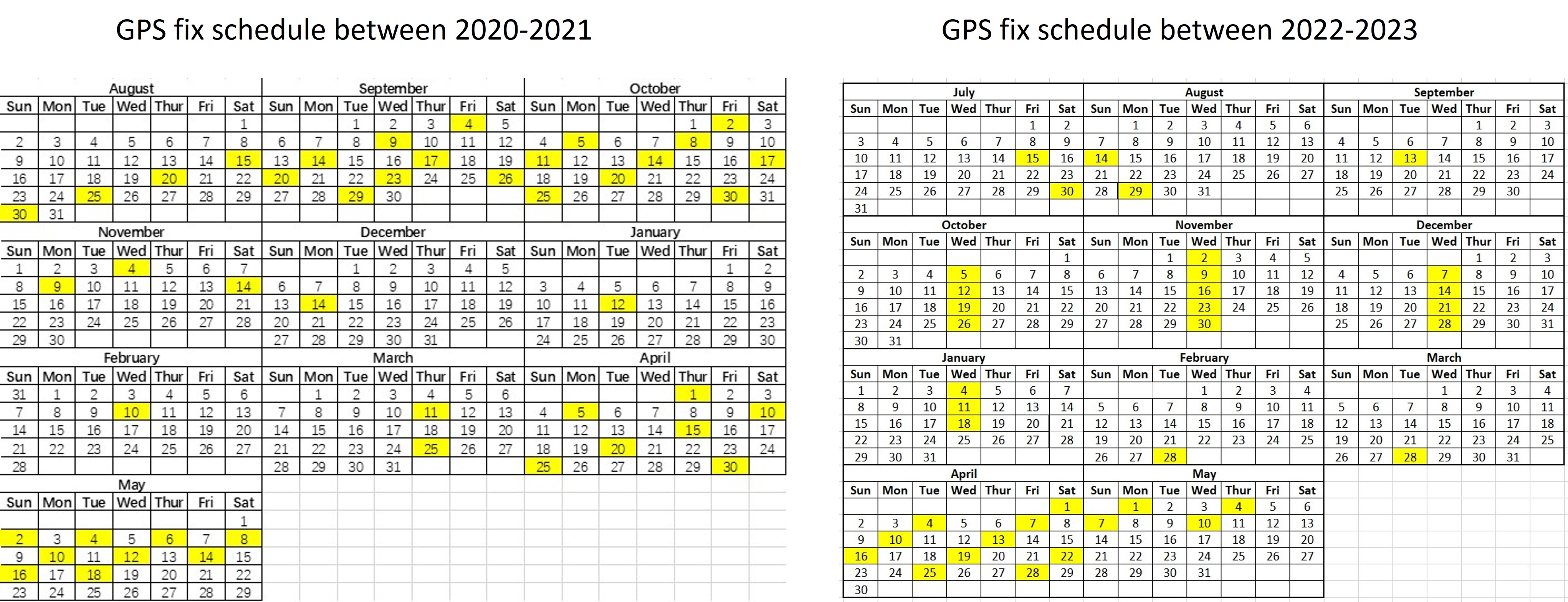

Supplement: Supplementary file 4 — Supplementary Material 4: Figure S2. The GPS logger schedule for 2020–2021 (left) and 2022–2023 (right), in which the yellow-highlighted dates were scheduled to have a geolocation fix at 00:00 or 01:00 GMT, equivalent to the local time at 5:00 – 7:00 (GMT+6 to +8). [file 40462_2024_495_MOESM4_ESM.jpg]

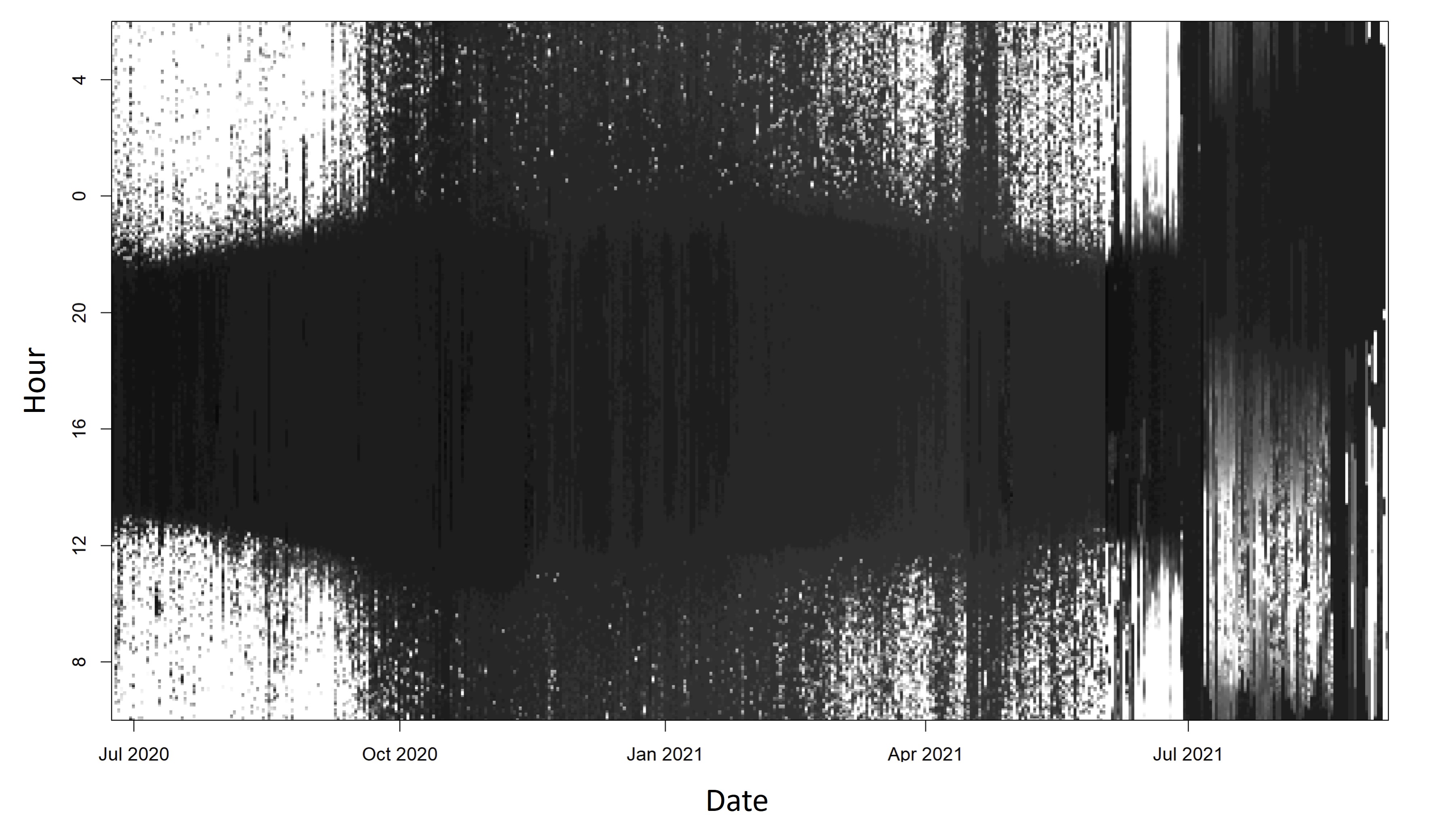

Supplement: Supplementary file 5 — Supplementary Material 5: Figure S3. The light data from the one light-level geolocator (GL484) in 2020–2021. The light-image indicates poor data quality during the non-breeding season of the tracked individual as an example, which applied for all individuals. [file 40462_2024_495_MOESM5_ESM.jpg]

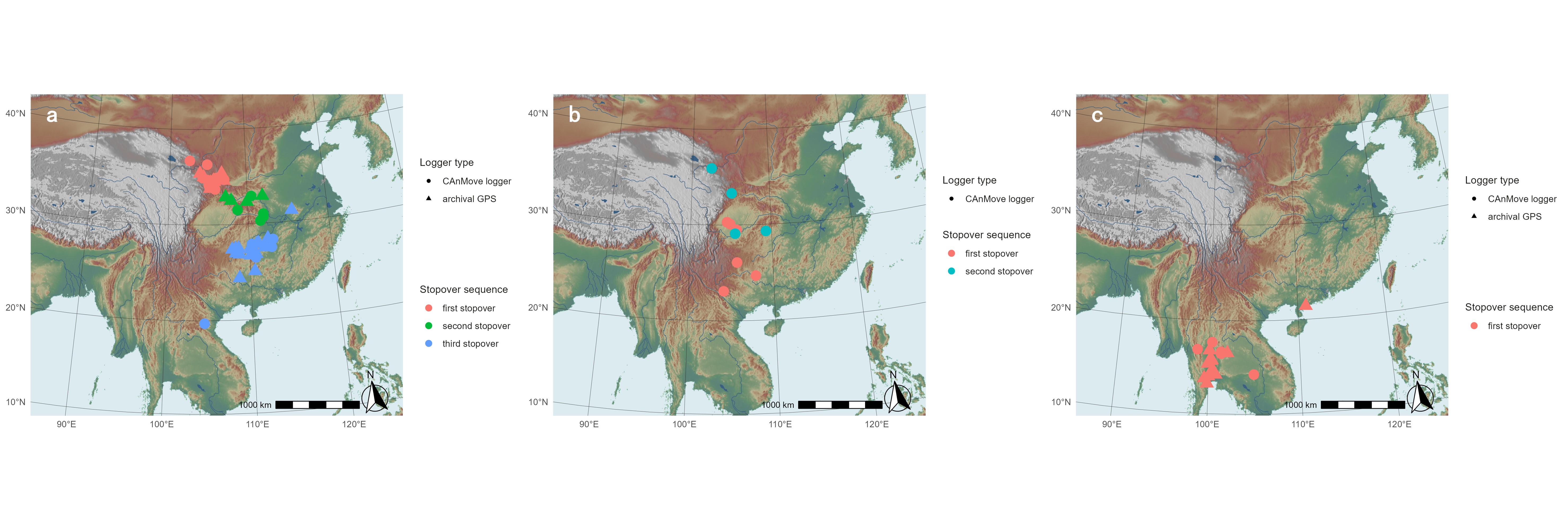

Supplement: Supplementary file 6 — Supplementary Material 6: Figure S4. Illustration of the main non-breeding stopovers of Siberian Rubythroats from the studied breeding population: a. Autumn long stopovers (> 3days) during migration; different colors represent three categorized stopover ranges, and different shape of points represent different origin of dataset. b. Spring long stopovers (> 2days) during migration; different colors represent whether it was the first or second time an individual had long stopovers. c. Wintering locations. [file 40462_2024_495_MOESM6_ESM.jpg]

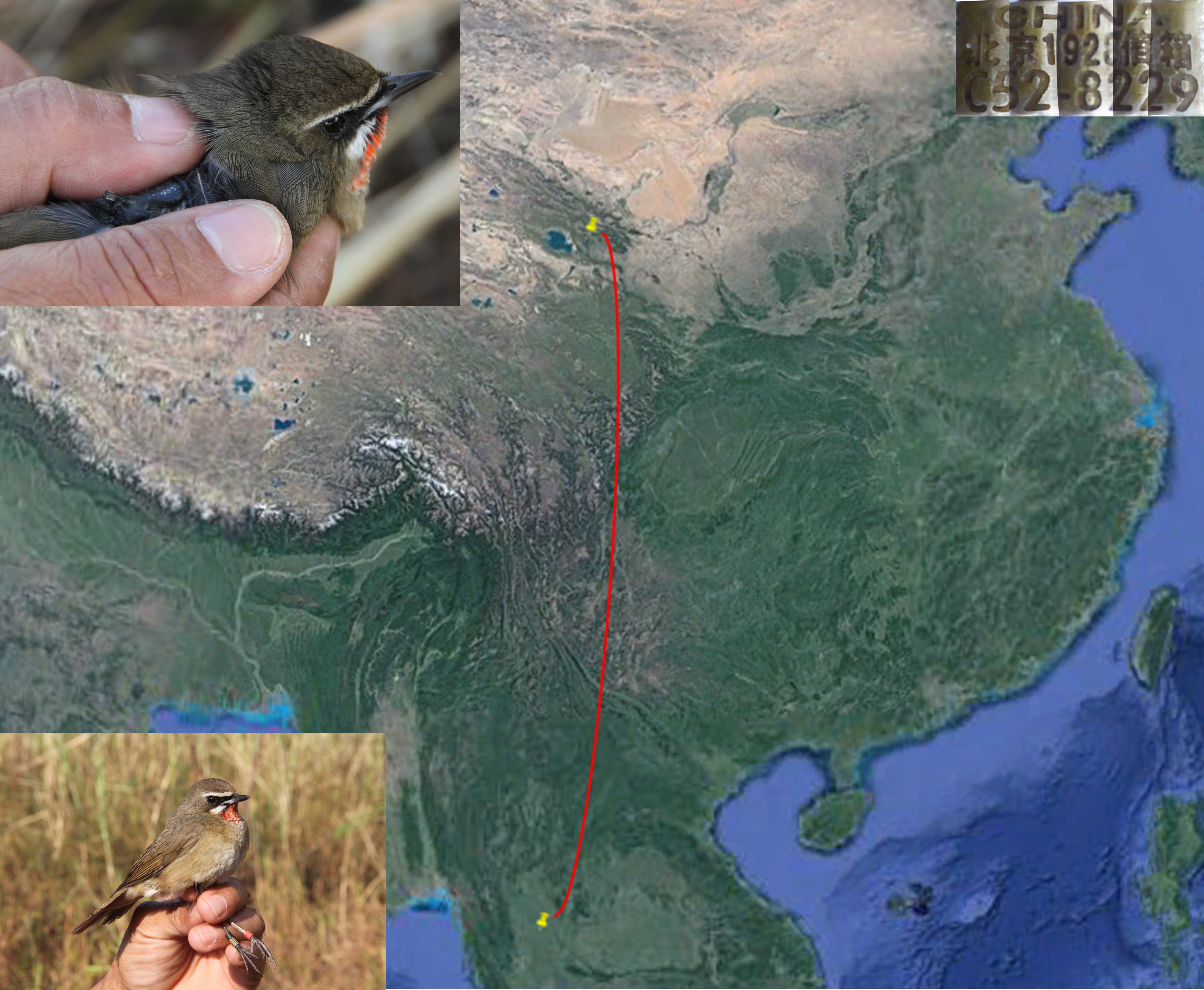

Supplement: Supplementary file 7 — Supplementary Material 7: Figure S5. The geolocation of the ringing recovery of one of the GL-tracked individuals (GL485, Ring number C52-8229) in Thailand in January 2021. [file 40462_2024_495_MOESM7_ESM.jpg]

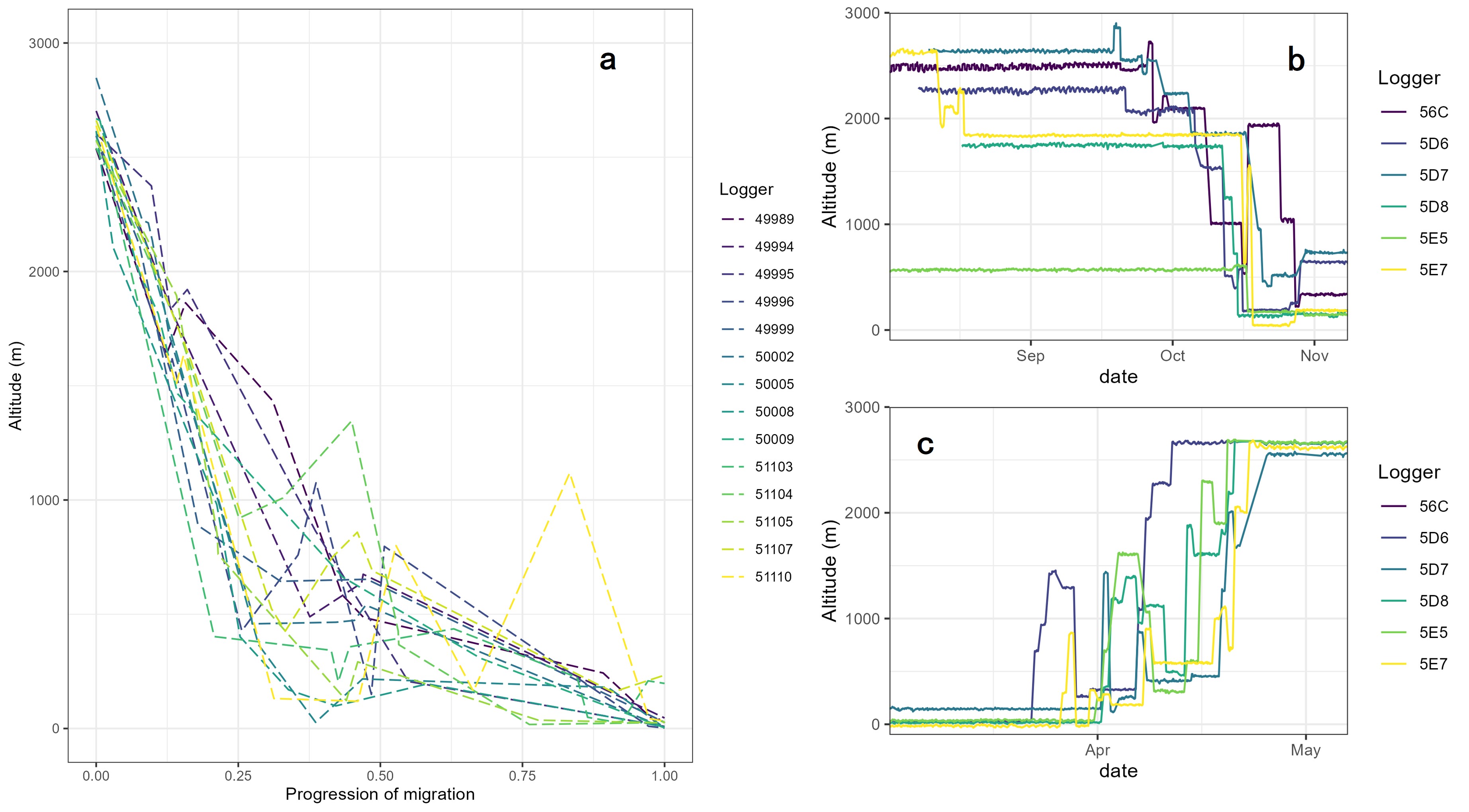

Supplement: Supplementary file 8 — Supplementary Material 8: Figure S6. The altitudinal movement of the a. archival GPS-tracked Siberian Rubythroats in autumn; b–c. CAnMove logger-tracked Siberian Rubythroats in both autumn and spring migration; each color represents one individual. 2. a. The autumn altitudinal movement of Siberian Rubythroat (n = 10) in 2020; the x-axis “Progression of migration” represents the percentage of cumulative distance that the bird had migrated against its total migration distance; b. The autumn elevational movement of Siberian Rubythroats (n = 6) between August 15th to November 20th, 2021. c. the spring elevational movement of Siberian Rubythroat (n = 5) between March 20th to May 20th, 2022. [file 40462_2024_495_MOESM8_ESM.jpg]

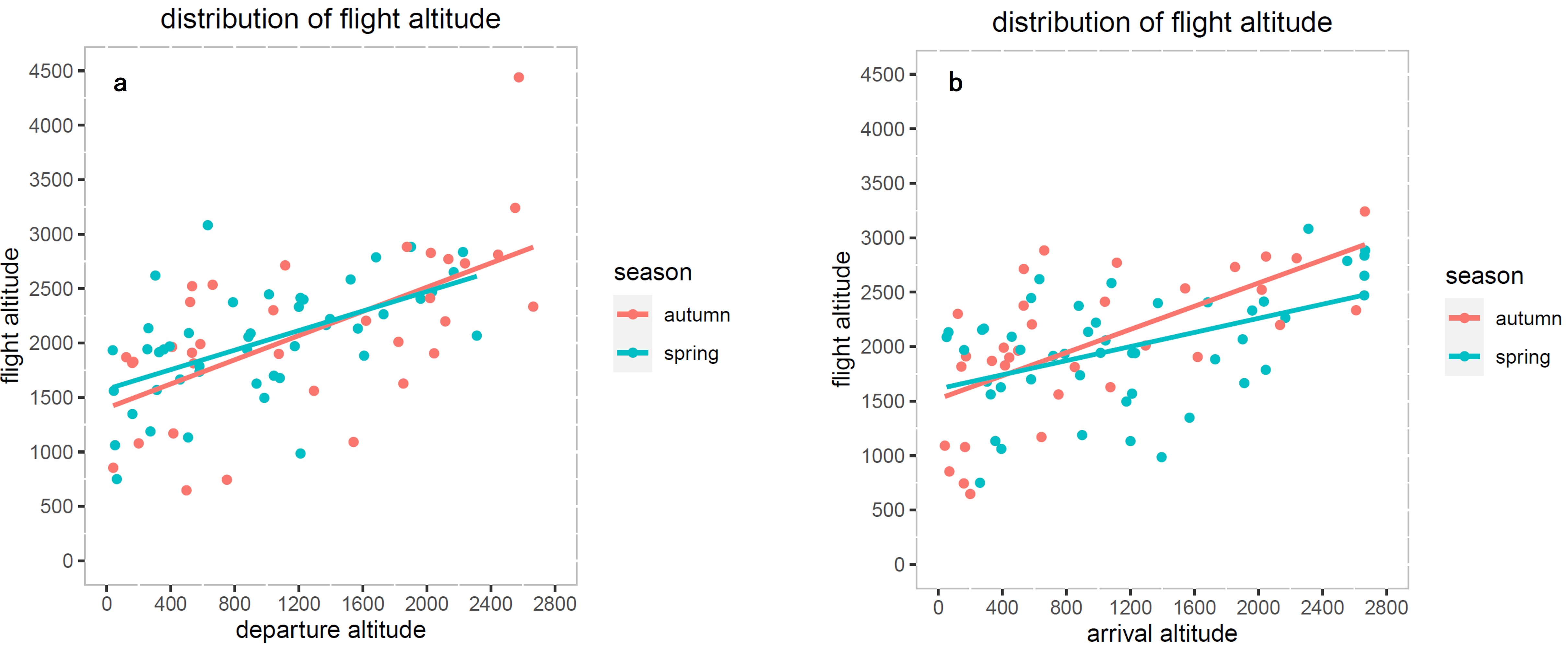

Supplement: Supplementary file 9 — Supplementary Material 9: Figure S7. The correlation between flight altitude (m a.s.l.) with a. the departure stopover elevation in spring (green) and autumn (red) and b. the arrival stopover elevation in spring (green) and autumn (red). A reference line y = x was added as a dashed line in each plot; along and below this line, flight events would be regarded as no/little flight height above ground. [file 40462_2024_495_MOESM9_ESM.jpg]

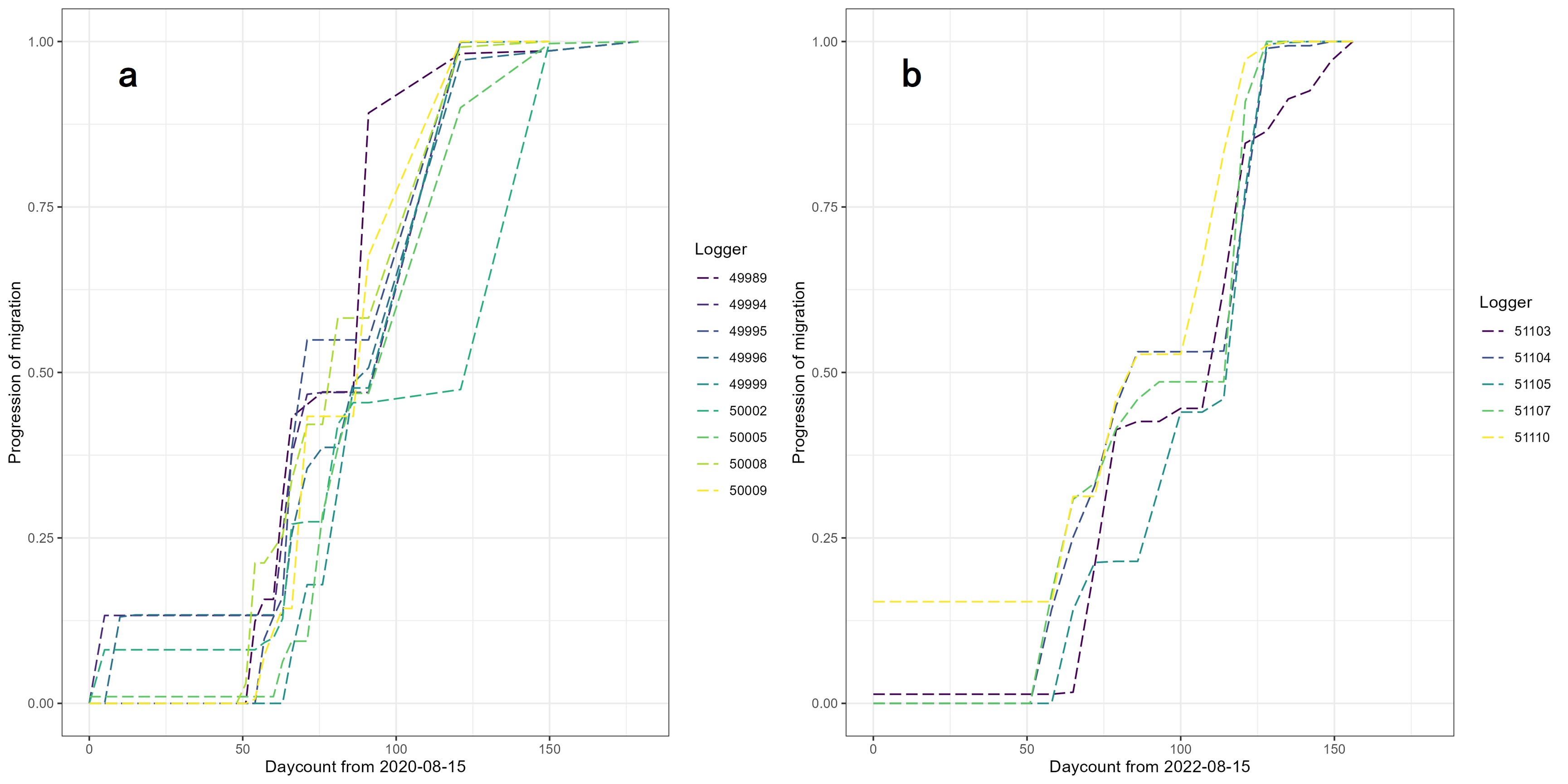

Supplement: Supplementary file 10 — Supplementary Material 10: Figure S8. The cumulative plots of autumn migration of Siberian Rubythroats in a. 2020 (n = 10) and b. 2022 (n = 5); the y-axis represents the percentage of cumulative distance that the bird had migrated against its total migration distance; each color represents one individual. [file 40462_2024_495_MOESM10_ESM.jpg]

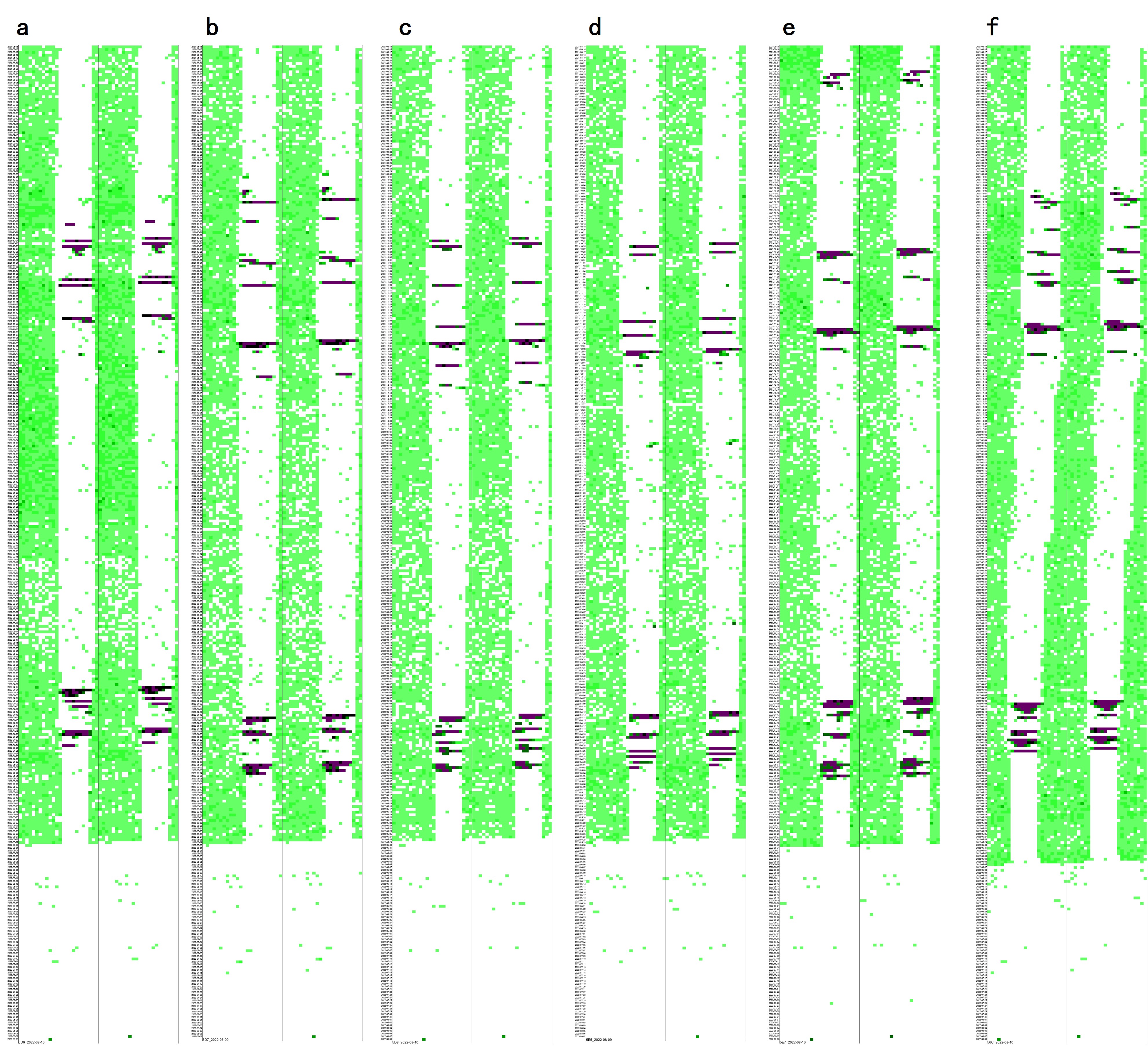

Supplement: Supplementary file 11 — Supplementary Material 11: Figure S9. The actograms of all CAnMove logger-tracked Siberian Rubythroats from the Qinghai breeding populations in 2021-2022: a–f represents actograms from individual 5D6, 5D7, 5D8, 5E5, 5E7, 56C, respectively. [file 40462_2024_495_MOESM11_ESM.jpg]

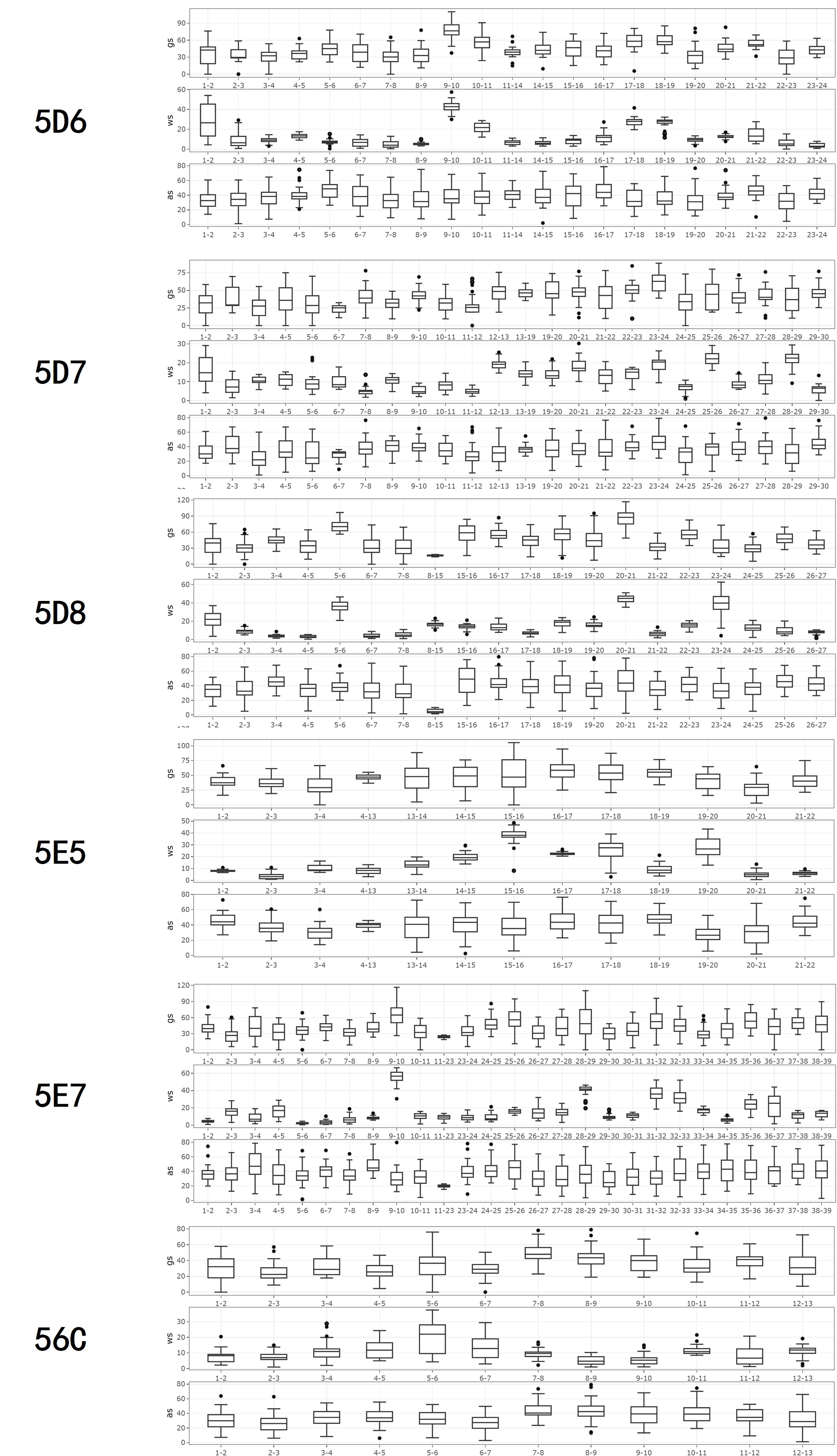

Supplement: Supplementary file 12 — Supplementary Material 12: Figure S10. The migration flight speed and wind speed of each flight event, estimated from the CAnMove logger dataset. The “gs” stands for ground speed, the “as” stands for air speed, and the “ws” stands for wind speed. [file 40462_2024_495_MOESM12_ESM.jpg]

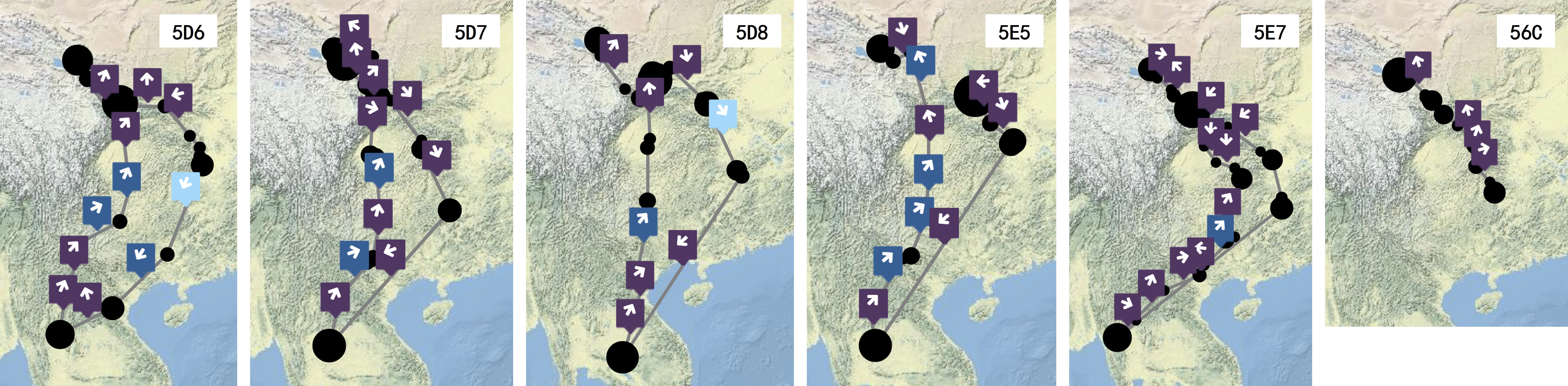

Supplement: Supplementary file 13 — Supplementary Material 13: Figure S11. The inferred migration routes and the inferred wind direction during each flight event. The arrows on migration paths represent the wind directions during the flight event. Lighter-colored arrows indicate greater alignment of the flight and wind direction during the flight event. [file 40462_2024_495_MOESM13_ESM.jpg]

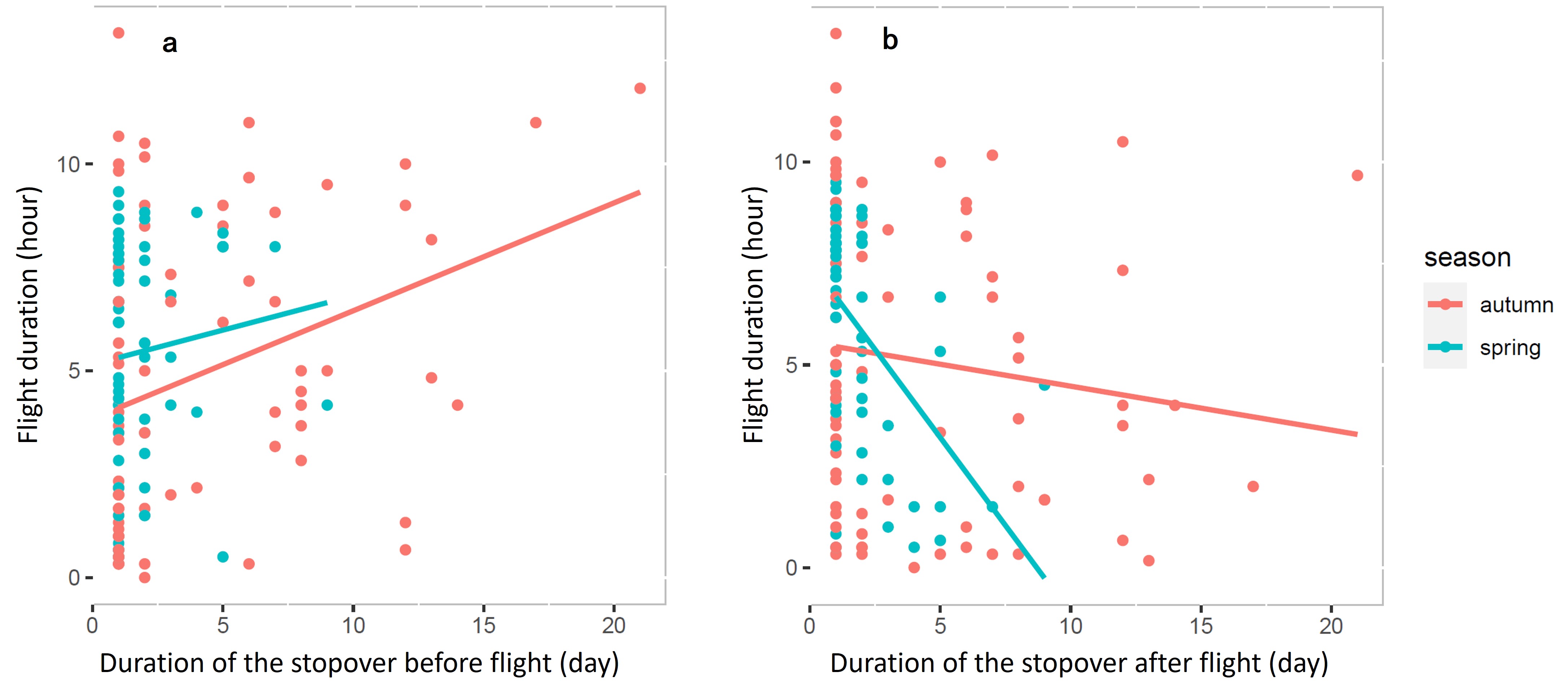

Supplement: Supplementary file 14 — Supplementary Material 14: Figure S12. The correlation between the flight duration and the stopover duration a. before or b. after each flight event (adjusted flight hours); red dots and line represents dataset from autumn, and green dots and line represents dataset from spring. [file 40462_2024_495_MOESM14_ESM.jpg]

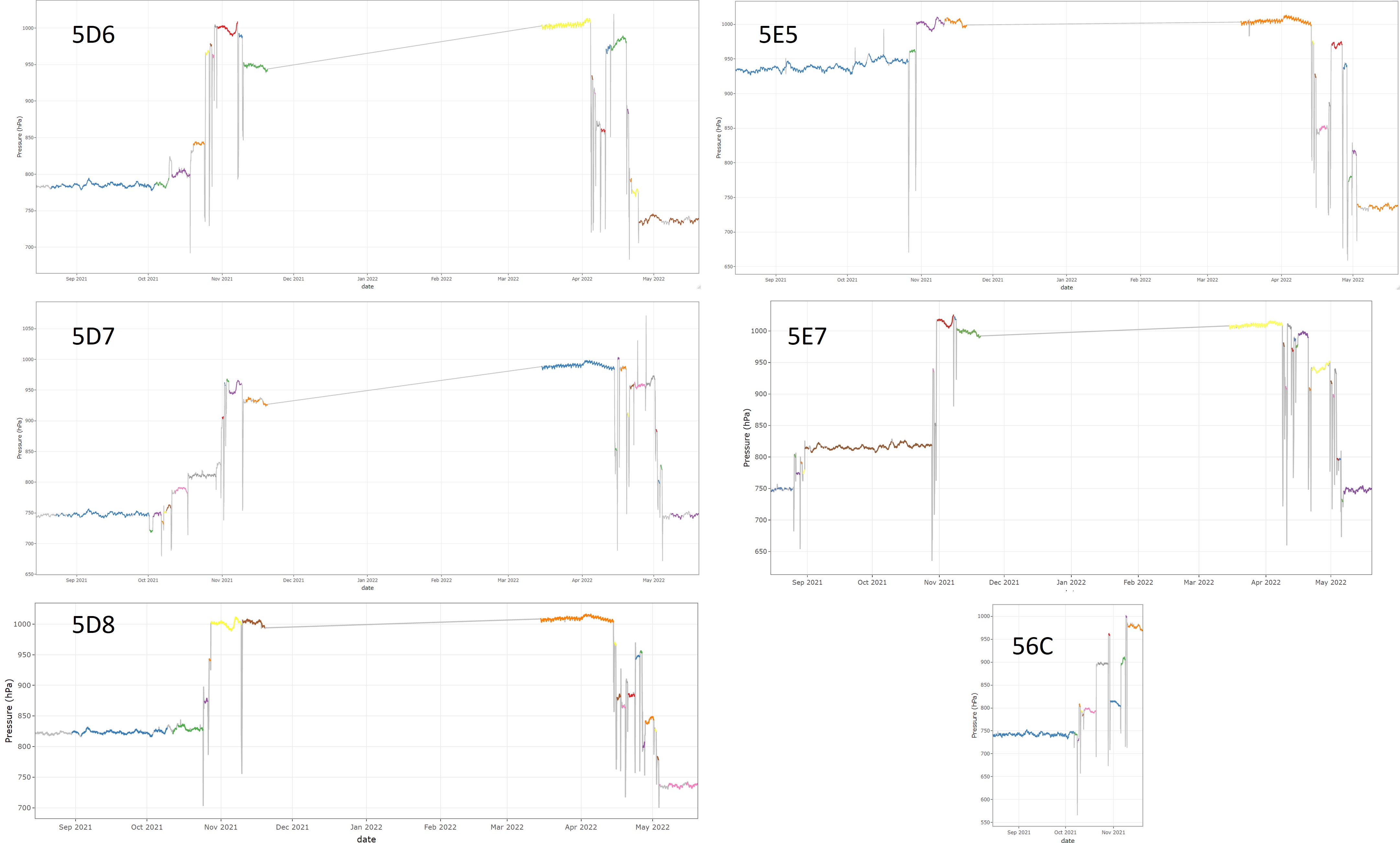

Supplement: Supplementary file 15 — Supplementary Material 15: Figure S13. The raw annual pressure data set from each CAnMove logger. The grey dash-line between November 20th, 2021 and March 15th, 2022 represents the lack of pressure data due to the data-collection settings. [file 40462_2024_495_MOESM15_ESM.jpg]
